# Supplementary material for: Estimated health benefits, costs, and cost-effectiveness of eliminating industrial trans-fatty acids in Australia: A modelling study
Source: PLoS Med. 2020 Nov 2;17(11):e1003407. doi: 10.1371/journal.pmed.1003407 (PMC7605626; doi:10.1371/journal.pmed.1003407)
Supplement: S1 Text — (DOCX) [file pmed.1003407.s002.docx]

**S1 Text - Study proposal**

**Background:** Ischemic heart disease (IHD) is the single most common cause of death in Australia, contributing to 12% of all deaths in 2016 [1]. Several environmental factors (including diet) influence the risk of IHD. A well-known dietary risk factor of IHD is a high intake of trans fatty acids (TFAs), a group of unsaturated fatty acids with one or more double bonds in trans configuration. A meta-analysis of prospective studies reported that for every 2 % of total energy (%E) from TFAs, the IHD risk increase by 23%. TFAs occur naturally at low levels in meat and milk from ruminants, but are also found in processed foods due to use of partially hydrogenated vegetable oils.

Recently, we estimated that industrial-derived TFAs contributes to 487 IHD deaths annually in Australia, equivalent to 1.5% of all IHD deaths [2]. Given the adverse effects of TFAs, the World Health Organization recommends to limit TFA intake to 1 %E and several countries have implemented strategies to reduce industrial derived TFA in the food supply [3, 4]. In addition to a reduction of IHD mortality, a reduction of TFA intake within the population can result in substantial savings in IHD-related health care costs for the society [5].

The risk of IHD as well as TFA intake differ within populations [5]. Recently, we found that TFA intake was highest in those with the least education and income[2] and given that IHD-burden is greater in these groups, it is possible that the greatest benefits from an elimination (e.g., by a nationwide ban) of industrially-derived TFA from the food supply would be among the most disadvantaged groups of the Australian population. In addition, information on TFA intake and the potential benefits of a TFA reduction in other subgroups (e.g., by sex, remoteness, and Aboriginals and Torres Strait Islanders vs. other Australians) could inform measures to reduce health inequalities in the Australian population.

**Hypotheses:** A nationwide ban of industrial TFAs would lead to decreased TFA intake and would result in reduced mortality, morbidity, and health care costs related to IHD, possibly with more pronounced benefits in certain subgroups of the Australian population.

**Specific Aim 1:** To estimate the potential effects on IHD and related health care costs of a nationwide ban of industrial trans-fatty acids (TFAs) in Australia.

**Specific Aim 2:** To examine potential inequalities of the effects on IHD and related health care costs, by estimating effects separately in subgroups by sex, socioeconomic status, remoteness, and Aboriginal status.

**Specific Aim 3:** To estimate the cost-effectiveness of a nationwide ban of industrial TFA.*

*Cost-effectiveness defined as the cost in Australian dollars per health-adjusted life year (HALY). These will be compared against the cost effectiveness ratios to WHO benchmarks, which define a cost effectiveness ratio <3×gross domestic product (GDP) per capita as cost effective, and <1×GDP per capita as highly cost effective.

**Methods:** Intake of TFA will be assessed using the 2011–2012 Australian National Nutrition and Physical Activity Survey. The IHD burden attributable to TFA will be calculated by comparing the current level of TFA intake to a counterfactual setting where consumption is lowered to a theoretical minimum distribution of 0.5% energy per day (corresponding to TFA intake only from non-industrial sources, e.g., dairy foods). Markov cohort models will used to estimate the impact on IHD burden in the Australian population and in subgroups (**Table**). Cost-effectiveness will be defined as the cost in Australian dollars per HALY. These will be compared against the cost effectiveness ratios to WHO benchmarks, which define a cost effectiveness ratio <3×gross domestic product (GDP) per capita as cost effective, and <1×GDP per capita as highly cost effective. Intervention effects will be modelled over three time intervals: 5y, 10y, and lifetime. Inequalities between subgroups will be evaluated by calculating concentration and slope indices [5, 6].

|  |
| --- |

**Refereneces**

1. Australian Bureau of Statistics. *Causes of Death, Australia, 2016, cat. no. 3303.0*. 2017 7 February 2018 [cited 2018 February 13]; Available from: http://www.abs.gov.au/ausstats/abs@.nsf/0/47E19CA15036B04BCA2577570014668B?Opendocument.

2. Wu, J.H., et al., *Contribution of Trans-Fatty Acid Intake to Coronary Heart Disease Burden in Australia: A Modelling Study.* Nutrients, 2017. **9**(1).

3. Uauy, R., et al., *WHO Scientific Update on trans fatty acids: summary and conclusions.* European Journal Of Clinical Nutrition, 2009. **63**: p. S68.

4. Downs, S.M., A.M. Thow, and S.R. Leeder, *The effectiveness of policies for reducing dietary trans fat: a systematic review of the evidence.* Bull World Health Organ, 2013. **91**(4): p. 262-9h.

5. Allen, K., et al., *Potential of trans fats policies to reduce socioeconomic inequalities in mortality from coronary heart disease in England: cost effectiveness modelling study.* BMJ, 2015. **351**: p. h4583.

6. Kakwani, N., A. Wagstaff, and E. van Doorslaer, *Socioeconomic inequalities in health: Measurement, computation, and statistical inference.* Journal of Econometrics, 1997. **77**(1): p. 87-103.

| **Table.** Subgroups, input data, and estimated outcomes | | | |
| --- | --- | --- | --- |
| **Data type** | **Parameter/Variable** | **Value, source or comment** |  |
| Subgroups | Sex | Men |  |
|  |  | Women |  |
|  | Socioeconomic status | SEIFA quintiles |  |
|  | Remoteness | Major cities |  |
|  |  | Inner regional |  |
|  |  | Other (outer regional, remote, and very remote) |  |
|  | Aboriginal status | Aboriginals and Torres Strait Islanders |  |
|  |  | Others |  |
| Input data | TFA intake | 2011–2012 Australian National Nutrition and Physical Activity Survey |  |
|  | TFA intake in Aboriginals and Torres Strait Islanders | Australian Aboriginal and Torres Strait Islander Health Survey (AATSIHS) |  |
|  | Population | ABS 2011 Census of Population and Housing |  |
|  | Mortality rate | ABS 2011 Census of Population and Housing |  |
|  | Subgroup mortality rate | Mortality inequalities in Australia 2009–2011 |  |
|  | IHD morbidity and mortality | GBD 2010 |  |
|  | Subgroup IHD rates | AIHW Cardiovascular disease web pages data tables |  |
|  | Health care costs | AIHW 2001 inflated to 2010 |  |
| Estimated outcomes | Averted IHD deaths | Calculated as the difference between current and counterfactual scenarios |  |
|  | HALYs gained | Calculated as the difference between current and counterfactual scenarios |  |
|  | IHD-related healthcare savings | Calculated as the difference between current and counterfactual scenarios |  |
|  | Cost-effectiveness | In Australian dollars per HALY gained* |  |
| ABS, Australian Bureau of Statistics; AIHW, Australian Institute of Health and Welfare; GBD, Global Burden of Disease; HALY, Health-adjusted life year; IHD, ischemic heart disese; SEIFA, Socio-Economic Indexes for Areas; TFA, trans fatty acid *These will be compared against the cost effectiveness ratios to WHO benchmarks, which define a cost effectiveness ratio <3×gross domestic product (GDP) per capita as cost effective, and <1×GDP per capita as highly cost effective. | | | |
