# Supplementary material for: Estimated health benefits, costs, and cost-effectiveness of eliminating industrial trans-fatty acids in Australia: A modelling study
Source: PLoS Med. 2020 Nov 2;17(11):e1003407. doi: 10.1371/journal.pmed.1003407 (PMC7605626; doi:10.1371/journal.pmed.1003407)
Supplement: S1 Table — (DOCX) [file pmed.1003407.s003.docx]

**S1 Table.** Total and subgroup-specific trans fatty intakes (%E) per age group, estimated in the 2011–2012 National Nutrition and Physical Activity Survey (NNPAS)

|  |  | Women | | |  | Men | | |
| --- | --- | --- | --- | --- | --- | --- | --- | --- |
| Population | Age (y) | N | Mean | SD |  | N | Mean | SD |
| Total population | 25-34 | 878 | 0.56 | 0.36 |  | 879 | 0.59 | 0.39 |
|  | 35-44 | 873 | 0.58 | 0.33 |  | 882 | 0.55 | 0.33 |
|  | 45-54 | 830 | 0.56 | 0.34 |  | 855 | 0.56 | 0.37 |
|  | 55-64 | 705 | 0.61 | 0.41 |  | 725 | 0.56 | 0.40 |
|  | 65-74 | 459 | 0.59 | 0.38 |  | 478 | 0.58 | 0.39 |
|  | 75+ | 307 | 0.65 | 0.41 |  | 384 | 0.64 | 0.42 |
|  |  |  |  |  |  |  |  |  |
| SEIFA Quintiles^1^ |  |  |  |  |  |  |  |  |
| 1 (most disadvantaged) | 25-34 | 153 | 0.63 | 0.46 |  | 150 | 0.60 | 0.41 |
|  | 35-44 | 137 | 0.59 | 0.33 |  | 133 | 0.58 | 0.40 |
|  | 45-54 | 117 | 0.54 | 0.36 |  | 138 | 0.54 | 0.36 |
|  | 55-64 | 151 | 0.65 | 0.53 |  | 132 | 0.65 | 0.48 |
|  | 65-74 | 96 | 0.57 | 0.35 |  | 116 | 0.58 | 0.44 |
|  | 75+ | 91 | 0.54 | 0.34 |  | 70 | 0.67 | 0.43 |
|  |  |  |  |  |  |  |  |  |
| 2 | 25-34 | 175 | 0.62 | 0.42 |  | 220 | 0.55 | 0.33 |
|  | 35-44 | 156 | 0.53 | 0.32 |  | 164 | 0.57 | 0.31 |
|  | 45-54 | 148 | 0.62 | 0.47 |  | 169 | 0.53 | 0.32 |
|  | 55-64 | 158 | 0.60 | 0.37 |  | 152 | 0.62 | 0.36 |
|  | 65-74 | 111 | 0.56 | 0.35 |  | 109 | 0.58 | 0.33 |
|  | 75+ | 85 | 0.70 | 0.46 |  | 61 | 0.69 | 0.36 |
|  |  |  |  |  |  |  |  |  |
| 3 | 25-34 | 216 | 0.52 | 0.33 |  | 176 | 0.58 | 0.35 |
|  | 35-44 | 204 | 0.59 | 0.36 |  | 173 | 0.61 | 0.34 |
|  | 45-54 | 171 | 0.62 | 0.40 |  | 198 | 0.55 | 0.35 |
|  | 55-64 | 147 | 0.50 | 0.31 |  | 137 | 0.59 | 0.38 |
|  | 65-74 | 97 | 0.66 | 0.47 |  | 84 | 0.60 | 0.35 |
|  | 75+ | 69 | 0.75 | 0.48 |  | 73 | 0.63 | 0.35 |
|  |  |  |  |  |  |  |  |  |
| 4 | 25-34 | 177 | 0.57 | 0.30 |  | 204 | 0.50 | 0.31 |
|  | 35-44 | 184 | 0.52 | 0.31 |  | 210 | 0.57 | 0.31 |
|  | 45-54 | 180 | 0.52 | 0.31 |  | 144 | 0.62 | 0.33 |
|  | 55-64 | 116 | 0.53 | 0.38 |  | 140 | 0.59 | 0.39 |
|  | 65-74 | 83 | 0.58 | 0.41 |  | 70 | 0.64 | 0.42 |
|  | 75+ | 69 | 0.64 | 0.38 |  | 56 | 0.68 | 0.49 |
|  |  |  |  |  |  |  |  |  |
| 5 (most advantaged) | 25-34 | 170 | 0.63 | 0.41 |  | 140 | 0.58 | 0.43 |
|  | 35-44 | 212 | 0.54 | 0.33 |  | 203 | 0.57 | 0.31 |
|  | 45-54 | 251 | 0.52 | 0.29 |  | 192 | 0.56 | 0.35 |
|  | 55-64 | 162 | 0.51 | 0.36 |  | 153 | 0.60 | 0.42 |
|  | 65-74 | 97 | 0.53 | 0.34 |  | 87 | 0.57 | 0.36 |
|  | 75+ | 74 | 0.57 | 0.42 |  | 52 | 0.59 | 0.42 |
|  |  |  |  |  |  |  |  |  |
| Remoteness |  |  |  |  |  |  |  |  |
| Major cities | 25-34 | 675 | 0.57 | 0.37 |  | 716 | 0.53 | 0.34 |
|  | 35-44 | 652 | 0.54 | 0.33 |  | 612 | 0.55 | 0.31 |
|  | 45-54 | 616 | 0.52 | 0.33 |  | 580 | 0.53 | 0.33 |
|  | 55-64 | 496 | 0.51 | 0.35 |  | 454 | 0.59 | 0.42 |
|  | 65-74 | 333 | 0.56 | 0.37 |  | 294 | 0.58 | 0.40 |
|  | 75+ | 273 | 0.62 | 0.39 |  | 214 | 0.61 | 0.41 |
|  |  |  |  |  |  |  |  |  |
| Inner regional | 25-34 | 142 | 0.65 | 0.45 |  | 120 | 0.66 | 0.44 |
|  | 35-44 | 161 | 0.59 | 0.32 |  | 184 | 0.62 | 0.32 |
|  | 45-54 | 178 | 0.66 | 0.40 |  | 162 | 0.61 | 0.33 |
|  | 55-64 | 151 | 0.70 | 0.52 |  | 169 | 0.62 | 0.32 |
|  | 65-74 | 113 | 0.61 | 0.41 |  | 121 | 0.56 | 0.32 |
|  | 75+ | 74 | 0.69 | 0.55 |  | 69 | 0.77 | 0.42 |
|  |  |  |  |  |  |  |  |  |
| Outer regional, | 25-34 | 91 | 0.68 | 0.47 |  | 54 | 0.68 | 0.38 |
| remote, & very remote | 35-44 | 87 | 0.56 | 0.37 |  | 74 | 0.63 | 0.37 |
|  | 45-54 | 50 | 0.73 | 0.39 |  | 89 | 0.72 | 0.39 |
|  | 55-64 | 38 | 0.64 | 0.43 |  | 80 | 0.60 | 0.40 |
|  | 65-74 | 28 | 0.72 | 0.31 |  | 98 | 0.64 | 0.40 |
|  | 75+ | 42 | 0.61 | 0.40 |  | 73 | 0.65 | 0.49 |
| ^1^Quintiles defined according to the Index of Relative Socio-Economic Disadvantage of the Socio-Economic Indexes for Areas (SEIFA). | | | | | | | | |
