# Supplementary material for: Estimated health benefits, costs, and cost-effectiveness of eliminating industrial trans-fatty acids in Australia: A modelling study
Source: PLoS Med. 2020 Nov 2;17(11):e1003407. doi: 10.1371/journal.pmed.1003407 (PMC7605626; doi:10.1371/journal.pmed.1003407)
Supplement: S2 Table — (DOCX) [file pmed.1003407.s004.docx]

**S2 Table.** Total and subgroup-specific population size per year of age

|  |  | SEIFA Quintile^2,3^ | | | | |  | Remoteness^3^ | | |
| --- | --- | --- | --- | --- | --- | --- | --- | --- | --- | --- |
| Age (y) | Total population^1^ | 1 | 2 | 3 | 4 | 5 |  | Major cities | Inner regional | Outer regional, remote, & very remote |
| 20 | 317,647 | 57,022 | 56,297 | 55,350 | 55,304 | 57,477 |  | 214,086 | 47,472 | 26,902 |
| 21 | 317,913 | 58,956 | 59,236 | 57,068 | 56,281 | 57,199 |  | 221,418 | 45,592 | 27,169 |
| 22 | 318,062 | 58,481 | 60,052 | 57,320 | 55,783 | 55,218 |  | 220,501 | 43,308 | 27,114 |
| 23 | 323,020 | 58,491 | 60,249 | 58,154 | 56,226 | 53,710 |  | 220,980 | 41,602 | 27,498 |
| 24 | 328,412 | 59,104 | 61,791 | 59,298 | 56,579 | 52,408 |  | 222,184 | 41,355 | 28,692 |
| 25 | 331,083 | 60,359 | 62,453 | 61,150 | 58,344 | 52,064 |  | 226,547 | 41,157 | 29,413 |
| 26 | 328,707 | 60,683 | 63,684 | 62,312 | 60,027 | 52,866 |  | 231,111 | 41,147 | 29,917 |
| 27 | 329,347 | 59,974 | 63,497 | 62,456 | 60,756 | 52,672 |  | 230,696 | 40,905 | 30,184 |
| 28 | 321,579 | 59,979 | 64,054 | 63,963 | 62,277 | 53,412 |  | 233,848 | 41,580 | 30,613 |
| 29 | 316,540 | 58,181 | 62,193 | 62,533 | 61,924 | 53,606 |  | 229,547 | 41,048 | 30,017 |
| 30 | 306,952 | 57,787 | 61,634 | 62,803 | 61,928 | 53,932 |  | 228,389 | 41,788 | 29,983 |
| 31 | 301,554 | 54,691 | 58,929 | 60,698 | 60,808 | 54,076 |  | 221,272 | 40,564 | 29,316 |
| 32 | 296,266 | 53,186 | 58,245 | 59,787 | 60,249 | 54,962 |  | 218,020 | 41,334 | 28,809 |
| 33 | 296,014 | 51,776 | 56,102 | 59,163 | 60,247 | 55,337 |  | 213,243 | 42,199 | 28,851 |
| 34 | 297,411 | 50,788 | 55,851 | 59,215 | 61,029 | 57,095 |  | 213,101 | 43,251 | 29,277 |
| 35 | 302,452 | 50,791 | 55,436 | 59,268 | 61,861 | 58,888 |  | 212,518 | 44,782 | 30,520 |
| 36 | 309,717 | 51,064 | 56,231 | 60,427 | 63,467 | 61,190 |  | 216,426 | 46,654 | 30,907 |
| 37 | 318,561 | 51,681 | 56,572 | 61,827 | 65,074 | 64,691 |  | 220,142 | 49,103 | 32,160 |
| 38 | 332,308 | 53,057 | 58,512 | 62,589 | 66,794 | 67,082 |  | 224,480 | 51,540 | 33,522 |
| 39 | 337,508 | 54,916 | 60,662 | 65,476 | 69,847 | 71,134 |  | 233,266 | 55,246 | 35,059 |
| 40 | 319,934 | 56,108 | 61,496 | 66,761 | 71,361 | 73,862 |  | 236,856 | 57,385 | 36,851 |
| 41 | 315,847 | 53,458 | 57,321 | 62,257 | 66,920 | 70,011 |  | 222,716 | 54,228 | 34,500 |
| 42 | 304,287 | 53,036 | 57,208 | 61,909 | 65,683 | 69,717 |  | 218,750 | 55,201 | 34,955 |
| 43 | 298,587 | 51,203 | 54,625 | 59,307 | 63,709 | 66,959 |  | 210,313 | 53,131 | 33,626 |
| 44 | 298,447 | 50,256 | 54,109 | 58,341 | 61,475 | 65,458 |  | 204,710 | 52,854 | 33,190 |
| 45 | 301,964 | 50,784 | 54,127 | 57,913 | 61,851 | 65,264 |  | 204,554 | 52,914 | 33,570 |
| 46 | 310,771 | 52,027 | 54,970 | 58,353 | 61,745 | 65,852 |  | 205,683 | 54,500 | 33,906 |
| 47 | 314,396 | 53,853 | 56,932 | 60,398 | 62,909 | 67,853 |  | 210,550 | 56,850 | 35,678 |
| 48 | 314,567 | 54,373 | 58,049 | 61,382 | 64,121 | 67,698 |  | 211,819 | 58,441 | 36,446 |
| 49 | 313,106 | 54,736 | 58,045 | 61,484 | 63,195 | 67,377 |  | 209,337 | 59,613 | 36,912 |
| 50 | 303,265 | 55,346 | 58,361 | 60,876 | 62,769 | 66,691 |  | 207,286 | 59,886 | 37,818 |
| 51 | 298,416 | 53,745 | 56,137 | 58,568 | 59,928 | 64,072 |  | 199,311 | 57,909 | 36,131 |
| 52 | 291,550 | 52,938 | 56,070 | 57,672 | 59,162 | 62,148 |  | 195,066 | 58,117 | 35,740 |
| 53 | 285,537 | 52,330 | 54,571 | 56,258 | 57,261 | 60,426 |  | 189,962 | 56,676 | 35,057 |
| 54 | 281,815 | 51,436 | 53,798 | 54,589 | 55,579 | 58,736 |  | 184,788 | 55,455 | 34,687 |
| 55 | 273,644 | 51,476 | 53,204 | 54,206 | 54,314 | 57,563 |  | 182,207 | 55,298 | 34,044 |
| 56 | 265,462 | 50,605 | 52,219 | 52,883 | 52,644 | 55,040 |  | 176,192 | 54,464 | 33,484 |
| 57 | 263,253 | 49,106 | 50,953 | 51,007 | 51,103 | 53,018 |  | 170,401 | 53,348 | 32,020 |
| 58 | 254,534 | 49,091 | 50,735 | 51,160 | 50,778 | 52,712 |  | 168,805 | 53,657 | 32,613 |
| 59 | 251,510 | 48,382 | 49,351 | 49,519 | 48,744 | 50,356 |  | 162,893 | 52,743 | 31,301 |
| 60 | 247,289 | 48,601 | 49,250 | 49,041 | 48,091 | 49,411 |  | 161,090 | 52,609 | 31,233 |
| 61 | 240,320 | 48,342 | 49,098 | 48,224 | 47,304 | 48,382 |  | 158,360 | 52,735 | 30,746 |
| 62 | 242,501 | 47,338 | 47,970 | 46,498 | 45,638 | 46,338 |  | 153,311 | 51,291 | 29,631 |
| 63 | 251,482 | 47,778 | 47,979 | 46,555 | 45,351 | 46,048 |  | 153,606 | 51,501 | 29,027 |
| 64 | 212,792 | 51,169 | 50,761 | 48,820 | 47,706 | 48,224 |  | 161,082 | 55,154 | 30,889 |
| 65 | 204,189 | 43,238 | 42,579 | 40,175 | 38,555 | 39,228 |  | 132,728 | 45,994 | 25,419 |
| 66 | 194,774 | 42,374 | 40,585 | 38,541 | 36,966 | 36,035 |  | 125,486 | 44,812 | 24,588 |
| 67 | 175,944 | 40,917 | 39,423 | 36,716 | 34,743 | 34,430 |  | 120,383 | 42,925 | 23,261 |
| 68 | 172,541 | 37,951 | 35,575 | 32,499 | 30,550 | 29,572 |  | 106,792 | 38,739 | 20,956 |
| 69 | 160,947 | 37,885 | 35,454 | 32,367 | 29,923 | 28,548 |  | 105,002 | 38,470 | 21,091 |
| 70 | 155,105 | 36,232 | 33,269 | 29,952 | 27,521 | 25,844 |  | 98,532 | 35,368 | 19,273 |
| 71 | 148,037 | 35,966 | 32,431 | 28,829 | 26,446 | 24,113 |  | 95,416 | 34,387 | 18,348 |
| 72 | 139,935 | 34,812 | 31,356 | 27,837 | 24,946 | 22,507 |  | 91,915 | 32,533 | 17,438 |
| 73 | 134,152 | 33,461 | 30,154 | 26,193 | 23,095 | 21,057 |  | 87,694 | 30,469 | 16,256 |
| 74 | 127,665 | 33,018 | 28,923 | 25,259 | 22,151 | 19,251 |  | 84,151 | 29,527 | 15,355 |
| 75 | 119,044 | 31,593 | 27,524 | 23,581 | 20,700 | 18,095 |  | 80,089 | 27,653 | 14,259 |
| 76 | 113,032 | 29,732 | 26,091 | 22,042 | 18,929 | 16,321 |  | 75,062 | 25,531 | 12,979 |
| 77 | 108,783 | 28,707 | 24,538 | 20,551 | 17,926 | 15,154 |  | 71,602 | 23,689 | 12,095 |
| 78 | 104,550 | 27,697 | 23,864 | 19,931 | 16,720 | 14,528 |  | 68,621 | 23,249 | 11,413 |
| 79 | 104,399 | 26,847 | 22,859 | 18,660 | 16,043 | 13,435 |  | 65,559 | 22,090 | 10,739 |
| 80 | 100,028 | 27,016 | 22,604 | 18,592 | 16,051 | 13,662 |  | 66,122 | 21,710 | 10,703 |
| 81 | 93,362 | 25,799 | 21,895 | 17,827 | 15,164 | 12,726 |  | 63,708 | 20,492 | 9,903 |
| 82 | 88,040 | 24,081 | 20,253 | 16,572 | 13,675 | 11,939 |  | 59,221 | 18,982 | 9,030 |
| 83 | 80,809 | 22,593 | 19,149 | 15,435 | 13,050 | 11,019 |  | 56,137 | 17,563 | 8,322 |
| 84 | 74,745 | 20,561 | 17,443 | 14,048 | 11,814 | 10,115 |  | 51,547 | 15,732 | 7,431 |
| 85 | 67,510 | 19,068 | 16,077 | 12,888 | 10,678 | 9,172 |  | 47,423 | 14,545 | 6,648 |
| 86 | 59,344 | 17,296 | 14,532 | 11,348 | 9,519 | 8,208 |  | 42,743 | 13,061 | 5,793 |
| 87 | 52,011 | 14,998 | 12,559 | 9,962 | 8,397 | 7,244 |  | 37,503 | 11,299 | 4,926 |
| 88 | 45,814 | 12,867 | 10,740 | 8,513 | 7,196 | 6,342 |  | 32,470 | 9,480 | 4,322 |
| 89 | 38,464 | 11,366 | 9,307 | 7,465 | 6,241 | 5,404 |  | 28,352 | 8,344 | 3,683 |
| 90 | 30,825 | 9,416 | 7,768 | 6,073 | 5,158 | 4,583 |  | 23,583 | 6,868 | 3,022 |
| 91 | 21,863 | 7,515 | 6,100 | 4,804 | 3,975 | 3,442 |  | 18,642 | 5,334 | 2,271 |
| 92 | 17,808 | 5,078 | 4,185 | 3,337 | 2,705 | 2,466 |  | 12,693 | 3,743 | 1,625 |
| 93 | 14,593 | 4,171 | 3,388 | 2,633 | 2,110 | 1,876 |  | 10,085 | 2,993 | 1,337 |
| 94 | 11,298 | 3,221 | 2,705 | 2,072 | 1,801 | 1,491 |  | 8,002 | 2,411 | 1,078 |
| 95 | 8,748 | 2,520 | 2,006 | 1,524 | 1,302 | 1,153 |  | 6,088 | 1,697 | 849 |
| 96 | 6,234 | 1,932 | 1,525 | 1,228 | 966 | 860 |  | 4,661 | 1,367 | 597 |
| 97 | 4,337 | 1,258 | 1,018 | 795 | 703 | 630 |  | 3,147 | 923 | 403 |
| 98 | 2,881 | 939 | 695 | 539 | 434 | 412 |  | 2,243 | 589 | 247 |
| 99 | 1,921 | 577 | 469 | 340 | 309 | 264 |  | 1,423 | 386 | 187 |
| 100 | 2,943 | 388 | 310 | 266 | 192 | 191 |  | 2,305 | 643 | 316 |
| ^1^Data retrieved from ABS report 3101.0 - Australian Demographic Statistics, Dec 2010 - TABLE 59. Estimated Resident Population By Single Year Of Age, Australia. ^2^Quintiles defined according to the Index of Relative Socio-Economic Disadvantage of the Socio-Economic Indexes for Areas (SEIFA). ^3^Data retrieved from Australian Bureau of Statistics, *2011 Census of Population and Housing*, TableBuilder. | | | | | | | | | | |
