# Supplementary material for: Estimated health benefits, costs, and cost-effectiveness of eliminating industrial trans-fatty acids in Australia: A modelling study
Source: PLoS Med. 2020 Nov 2;17(11):e1003407. doi: 10.1371/journal.pmed.1003407 (PMC7605626; doi:10.1371/journal.pmed.1003407)
Supplement: S3 Table — (DOCX) [file pmed.1003407.s005.docx]

**S3 Table.** Total and subgroup-specific mortality rate (deaths per 100,000) per year of age

|  |  |  | SEIFA Quintile^2,3^ | | | | |  | Remoteness^3^ | | |  |
| --- | --- | --- | --- | --- | --- | --- | --- | --- | --- | --- | --- | --- |
| Sex | Age (y) | Total population^1^ | 1 | 2 | 3 | 4 | 5 |  | Major cities | Inner regional | Outer regional, remote, & very remote |  |
| Women | 20 | 26 | 32 | 31 | 24 | 20 | 21 |  | 20 | 34 | 50 |  |
|  | 21 | 27 | 33 | 31 | 24 | 21 | 21 |  | 20 | 35 | 51 |  |
|  | 22 | 27 | 33 | 32 | 24 | 21 | 22 |  | 21 | 35 | 52 |  |
|  | 23 | 27 | 34 | 32 | 25 | 21 | 22 |  | 21 | 36 | 52 |  |
|  | 24 | 28 | 34 | 33 | 25 | 22 | 22 |  | 21 | 36 | 53 |  |
|  | 25 | 28 | 39 | 34 | 28 | 22 | 20 |  | 24 | 34 | 45 |  |
|  | 26 | 29 | 40 | 35 | 29 | 23 | 21 |  | 25 | 36 | 46 |  |
|  | 27 | 30 | 42 | 36 | 30 | 24 | 22 |  | 26 | 37 | 48 |  |
|  | 28 | 32 | 44 | 37 | 31 | 25 | 22 |  | 27 | 39 | 50 |  |
|  | 29 | 33 | 46 | 39 | 32 | 26 | 23 |  | 28 | 40 | 52 |  |
|  | 30 | 35 | 48 | 41 | 34 | 27 | 25 |  | 30 | 42 | 54 |  |
|  | 31 | 36 | 50 | 43 | 35 | 29 | 26 |  | 31 | 44 | 57 |  |
|  | 32 | 39 | 53 | 46 | 38 | 31 | 27 |  | 33 | 47 | 61 |  |
|  | 33 | 42 | 57 | 49 | 40 | 33 | 29 |  | 36 | 50 | 65 |  |
|  | 34 | 45 | 62 | 53 | 44 | 36 | 32 |  | 38 | 55 | 71 |  |
|  | 35 | 49 | 67 | 58 | 48 | 39 | 35 |  | 42 | 60 | 77 |  |
|  | 36 | 54 | 74 | 63 | 52 | 42 | 38 |  | 46 | 65 | 84 |  |
|  | 37 | 59 | 81 | 69 | 57 | 46 | 41 |  | 50 | 71 | 92 |  |
|  | 38 | 64 | 88 | 76 | 62 | 51 | 45 |  | 55 | 78 | 100 |  |
|  | 39 | 70 | 96 | 82 | 68 | 55 | 49 |  | 59 | 84 | 109 |  |
|  | 40 | 75 | 104 | 89 | 73 | 60 | 53 |  | 65 | 92 | 118 |  |
|  | 41 | 82 | 112 | 97 | 79 | 65 | 58 |  | 70 | 99 | 128 |  |
|  | 42 | 89 | 122 | 105 | 86 | 70 | 63 |  | 76 | 108 | 139 |  |
|  | 43 | 96 | 132 | 114 | 93 | 76 | 68 |  | 82 | 117 | 151 |  |
|  | 44 | 104 | 144 | 123 | 102 | 83 | 74 |  | 89 | 127 | 164 |  |
|  | 45 | 114 | 149 | 130 | 112 | 97 | 82 |  | 105 | 125 | 141 |  |
|  | 46 | 124 | 162 | 141 | 122 | 106 | 90 |  | 114 | 136 | 153 |  |
|  | 47 | 135 | 177 | 154 | 133 | 115 | 98 |  | 125 | 149 | 167 |  |
|  | 48 | 147 | 193 | 168 | 145 | 126 | 107 |  | 136 | 162 | 183 |  |
|  | 49 | 161 | 210 | 183 | 158 | 137 | 116 |  | 148 | 177 | 199 |  |
|  | 50 | 175 | 229 | 199 | 172 | 149 | 127 |  | 161 | 192 | 217 |  |
|  | 51 | 190 | 248 | 217 | 187 | 162 | 138 |  | 175 | 209 | 235 |  |
|  | 52 | 206 | 269 | 235 | 203 | 176 | 149 |  | 190 | 227 | 255 |  |
|  | 53 | 223 | 291 | 254 | 220 | 190 | 161 |  | 205 | 245 | 276 |  |
|  | 54 | 240 | 314 | 274 | 237 | 205 | 174 |  | 221 | 264 | 297 |  |
|  | 55 | 259 | 338 | 295 | 255 | 221 | 187 |  | 238 | 284 | 320 |  |
|  | 56 | 279 | 364 | 318 | 275 | 238 | 202 |  | 257 | 306 | 345 |  |
|  | 57 | 301 | 393 | 343 | 296 | 257 | 218 |  | 277 | 331 | 373 |  |
|  | 58 | 326 | 426 | 371 | 321 | 278 | 236 |  | 300 | 358 | 404 |  |
|  | 59 | 355 | 463 | 404 | 349 | 303 | 257 |  | 327 | 390 | 439 |  |
|  | 60 | 386 | 505 | 440 | 381 | 330 | 280 |  | 356 | 425 | 479 |  |
|  | 61 | 422 | 551 | 480 | 416 | 360 | 305 |  | 389 | 464 | 522 |  |
|  | 62 | 460 | 601 | 524 | 453 | 393 | 333 |  | 424 | 506 | 570 |  |
|  | 63 | 501 | 655 | 571 | 494 | 428 | 363 |  | 462 | 551 | 621 |  |
|  | 64 | 546 | 713 | 622 | 538 | 466 | 395 |  | 503 | 600 | 676 |  |
|  | 65 | 595 | 648 | 630 | 584 | 569 | 519 |  | 577 | 617 | 644 |  |
|  | 66 | 651 | 710 | 690 | 640 | 623 | 568 |  | 632 | 676 | 705 |  |
|  | 67 | 718 | 782 | 761 | 705 | 687 | 626 |  | 697 | 745 | 777 |  |
|  | 68 | 798 | 870 | 846 | 784 | 764 | 696 |  | 775 | 828 | 864 |  |
|  | 69 | 896 | 976 | 949 | 879 | 857 | 781 |  | 869 | 929 | 969 |  |
|  | 70 | 1,009 | 1,100 | 1,069 | 991 | 965 | 880 |  | 980 | 1,047 | 1,093 |  |
|  | 71 | 1,139 | 1,241 | 1,206 | 1,118 | 1,089 | 993 |  | 1,105 | 1,182 | 1,233 |  |
|  | 72 | 1,281 | 1,396 | 1,357 | 1,258 | 1,225 | 1,117 |  | 1,243 | 1,329 | 1,387 |  |
|  | 73 | 1,432 | 1,560 | 1,517 | 1,406 | 1,370 | 1,249 |  | 1,390 | 1,486 | 1,550 |  |
|  | 74 | 1,591 | 1,733 | 1,685 | 1,562 | 1,521 | 1,387 |  | 1,544 | 1,651 | 1,722 |  |
|  | 75 | 1,763 | 1,921 | 1,867 | 1,731 | 1,686 | 1,537 |  | 1,711 | 1,829 | 1,908 |  |
|  | 76 | 1,957 | 2,132 | 2,073 | 1,921 | 1,871 | 1,706 |  | 1,899 | 2,030 | 2,118 |  |
|  | 77 | 2,184 | 2,380 | 2,314 | 2,144 | 2,089 | 1,904 |  | 2,120 | 2,266 | 2,364 |  |
|  | 78 | 2,462 | 2,683 | 2,608 | 2,417 | 2,355 | 2,147 |  | 2,389 | 2,555 | 2,665 |  |
|  | 79 | 2,804 | 3,055 | 2,970 | 2,753 | 2,682 | 2,444 |  | 2,721 | 2,909 | 3,035 |  |
|  | 80 | 3,216 | 3,504 | 3,407 | 3,157 | 3,076 | 2,804 |  | 3,121 | 3,337 | 3,481 |  |
|  | 81 | 3,704 | 4,036 | 3,924 | 3,637 | 3,543 | 3,229 |  | 3,595 | 3,843 | 4,009 |  |
|  | 82 | 4,271 | 4,653 | 4,525 | 4,193 | 4,085 | 3,724 |  | 4,145 | 4,432 | 4,623 |  |
|  | 83 | 4,916 | 5,356 | 5,207 | 4,826 | 4,701 | 4,286 |  | 4,770 | 5,100 | 5,321 |  |
|  | 84 | 5,641 | 6,145 | 5,975 | 5,538 | 5,395 | 4,918 |  | 5,474 | 5,852 | 6,105 |  |
|  | 85 | 6,458 | 6,347 | 6,567 | 6,537 | 6,463 | 6,337 |  | 6,266 | 6,822 | 7,050 |  |
|  | 86 | 7,384 | 7,258 | 7,509 | 7,475 | 7,390 | 7,246 |  | 7,165 | 7,800 | 8,061 |  |
|  | 87 | 8,439 | 8,295 | 8,582 | 8,543 | 8,446 | 8,281 |  | 8,189 | 8,915 | 9,214 |  |
|  | 88 | 9,649 | 9,484 | 9,812 | 9,767 | 9,657 | 9,469 |  | 9,363 | 10,193 | 10,534 |  |
|  | 89 | 11,030 | 10,841 | 11,216 | 11,165 | 11,039 | 10,823 |  | 10,702 | 11,652 | 12,042 |  |
|  | 90 | 12,579 | 12,363 | 12,791 | 12,733 | 12,589 | 12,344 |  | 12,206 | 13,288 | 13,733 |  |
|  | 91 | 14,283 | 14,038 | 14,524 | 14,458 | 14,295 | 14,015 |  | 13,859 | 15,088 | 15,593 |  |
|  | 92 | 16,112 | 15,836 | 16,384 | 16,309 | 16,125 | 15,810 |  | 15,634 | 17,020 | 17,590 |  |
|  | 93 | 18,021 | 17,712 | 18,325 | 18,242 | 18,036 | 17,684 |  | 17,486 | 19,037 | 19,674 |  |
|  | 94 | 20,000 | 19,657 | 20,338 | 20,245 | 20,017 | 19,626 |  | 19,406 | 21,128 | 21,835 |  |
|  | 95 | 22,100 | 21,721 | 22,473 | 22,371 | 22,118 | 21,686 |  | 21,444 | 23,346 | 24,127 |  |
|  | 96 | 24,400 | 23,982 | 24,812 | 24,699 | 24,420 | 23,943 |  | 23,675 | 25,776 | 26,638 |  |
|  |  |  |  |  |  |  |  |  |  |  |  |  |
| Men | 20 | 65 | 86 | 72 | 65 | 54 | 43 |  | 50 | 87 | 115 |  |
|  | 21 | 65 | 85 | 72 | 64 | 54 | 43 |  | 50 | 87 | 114 |  |
|  | 22 | 64 | 84 | 71 | 63 | 53 | 42 |  | 49 | 85 | 113 |  |
|  | 23 | 64 | 84 | 70 | 63 | 53 | 42 |  | 49 | 85 | 112 |  |
|  | 24 | 64 | 85 | 71 | 64 | 53 | 43 |  | 50 | 86 | 114 |  |
|  | 25 | 66 | 92 | 75 | 66 | 53 | 43 |  | 56 | 80 | 101 |  |
|  | 26 | 68 | 95 | 78 | 68 | 54 | 44 |  | 58 | 83 | 105 |  |
|  | 27 | 71 | 100 | 81 | 71 | 57 | 46 |  | 61 | 86 | 109 |  |
|  | 28 | 75 | 105 | 85 | 75 | 60 | 49 |  | 64 | 91 | 115 |  |
|  | 29 | 79 | 111 | 90 | 79 | 63 | 52 |  | 68 | 96 | 122 |  |
|  | 30 | 84 | 118 | 96 | 84 | 67 | 55 |  | 72 | 102 | 130 |  |
|  | 31 | 90 | 125 | 102 | 89 | 72 | 58 |  | 76 | 109 | 138 |  |
|  | 32 | 95 | 133 | 108 | 95 | 76 | 62 |  | 81 | 115 | 146 |  |
|  | 33 | 100 | 140 | 114 | 100 | 80 | 65 |  | 85 | 121 | 154 |  |
|  | 34 | 105 | 147 | 120 | 105 | 84 | 69 |  | 89 | 127 | 161 |  |
|  | 35 | 110 | 154 | 125 | 110 | 88 | 72 |  | 94 | 133 | 169 |  |
|  | 36 | 115 | 161 | 131 | 115 | 92 | 75 |  | 98 | 140 | 177 |  |
|  | 37 | 121 | 169 | 138 | 120 | 97 | 79 |  | 103 | 146 | 186 |  |
|  | 38 | 127 | 178 | 145 | 126 | 101 | 83 |  | 108 | 154 | 195 |  |
|  | 39 | 134 | 187 | 152 | 133 | 107 | 87 |  | 114 | 162 | 205 |  |
|  | 40 | 141 | 198 | 161 | 141 | 113 | 92 |  | 120 | 171 | 217 |  |
|  | 41 | 150 | 211 | 171 | 150 | 120 | 98 |  | 128 | 182 | 231 |  |
|  | 42 | 161 | 226 | 184 | 161 | 129 | 105 |  | 137 | 195 | 248 |  |
|  | 43 | 174 | 243 | 198 | 173 | 139 | 113 |  | 148 | 211 | 267 |  |
|  | 44 | 188 | 264 | 214 | 187 | 150 | 123 |  | 160 | 228 | 289 |  |
|  | 45 | 204 | 275 | 230 | 204 | 174 | 136 |  | 187 | 222 | 253 |  |
|  | 46 | 222 | 298 | 249 | 222 | 189 | 148 |  | 203 | 241 | 274 |  |
|  | 47 | 240 | 323 | 270 | 240 | 205 | 160 |  | 220 | 261 | 297 |  |
|  | 48 | 259 | 348 | 291 | 258 | 220 | 172 |  | 237 | 281 | 320 |  |
|  | 49 | 278 | 374 | 313 | 278 | 237 | 185 |  | 254 | 302 | 344 |  |
|  | 50 | 298 | 401 | 335 | 298 | 254 | 199 |  | 273 | 324 | 369 |  |
|  | 51 | 320 | 431 | 360 | 320 | 273 | 214 |  | 293 | 348 | 396 |  |
|  | 52 | 345 | 464 | 388 | 345 | 294 | 230 |  | 316 | 375 | 427 |  |
|  | 53 | 374 | 503 | 420 | 373 | 318 | 249 |  | 342 | 406 | 463 |  |
|  | 54 | 406 | 546 | 457 | 406 | 346 | 271 |  | 371 | 441 | 503 |  |
|  | 55 | 442 | 595 | 497 | 442 | 377 | 295 |  | 404 | 480 | 547 |  |
|  | 56 | 481 | 647 | 541 | 481 | 410 | 321 |  | 440 | 523 | 596 |  |
|  | 57 | 523 | 704 | 589 | 523 | 446 | 349 |  | 479 | 568 | 648 |  |
|  | 58 | 567 | 763 | 638 | 567 | 483 | 378 |  | 519 | 616 | 702 |  |
|  | 59 | 614 | 826 | 691 | 613 | 523 | 409 |  | 561 | 667 | 760 |  |
|  | 60 | 665 | 894 | 748 | 664 | 567 | 443 |  | 608 | 723 | 823 |  |
|  | 61 | 723 | 972 | 813 | 722 | 616 | 482 |  | 661 | 785 | 894 |  |
|  | 62 | 789 | 1,061 | 887 | 788 | 672 | 526 |  | 721 | 857 | 976 |  |
|  | 63 | 867 | 1,166 | 975 | 866 | 739 | 578 |  | 793 | 942 | 1,073 |  |
|  | 64 | 958 | 1,288 | 1,077 | 956 | 816 | 638 |  | 876 | 1,040 | 1,185 |  |
|  | 65 | 1,061 | 1,185 | 1,132 | 1,061 | 987 | 880 |  | 1,029 | 1,100 | 1,131 |  |
|  | 66 | 1,178 | 1,316 | 1,257 | 1,178 | 1,096 | 977 |  | 1,142 | 1,221 | 1,255 |  |
|  | 67 | 1,308 | 1,461 | 1,396 | 1,308 | 1,216 | 1,085 |  | 1,268 | 1,356 | 1,393 |  |
|  | 68 | 1,450 | 1,619 | 1,547 | 1,449 | 1,348 | 1,202 |  | 1,405 | 1,503 | 1,544 |  |
|  | 69 | 1,604 | 1,792 | 1,712 | 1,604 | 1,492 | 1,331 |  | 1,555 | 1,663 | 1,709 |  |
|  | 70 | 1,775 | 1,983 | 1,894 | 1,775 | 1,651 | 1,472 |  | 1,720 | 1,840 | 1,891 |  |
|  | 71 | 1,964 | 2,194 | 2,096 | 1,964 | 1,826 | 1,629 |  | 1,904 | 2,036 | 2,092 |  |
|  | 72 | 2,175 | 2,430 | 2,321 | 2,175 | 2,023 | 1,804 |  | 2,109 | 2,255 | 2,317 |  |
|  | 73 | 2,413 | 2,695 | 2,575 | 2,413 | 2,244 | 2,002 |  | 2,339 | 2,502 | 2,571 |  |
|  | 74 | 2,683 | 2,997 | 2,863 | 2,683 | 2,495 | 2,225 |  | 2,600 | 2,781 | 2,858 |  |
|  | 75 | 2,990 | 3,340 | 3,191 | 2,990 | 2,781 | 2,480 |  | 2,898 | 3,100 | 3,185 |  |
|  | 76 | 3,343 | 3,734 | 3,567 | 3,343 | 3,108 | 2,773 |  | 3,240 | 3,465 | 3,561 |  |
|  | 77 | 3,750 | 4,188 | 4,001 | 3,749 | 3,487 | 3,110 |  | 3,635 | 3,887 | 3,994 |  |
|  | 78 | 4,222 | 4,715 | 4,504 | 4,221 | 3,925 | 3,501 |  | 4,092 | 4,376 | 4,497 |  |
|  | 79 | 4,764 | 5,321 | 5,084 | 4,764 | 4,430 | 3,952 |  | 4,618 | 4,939 | 5,075 |  |
|  | 80 | 5,383 | 6,013 | 5,744 | 5,383 | 5,006 | 4,465 |  | 5,218 | 5,580 | 5,735 |  |
|  | 81 | 6,082 | 6,793 | 6,490 | 6,081 | 5,655 | 5,044 |  | 5,895 | 6,304 | 6,479 |  |
|  | 82 | 6,861 | 7,663 | 7,321 | 6,860 | 6,380 | 5,690 |  | 6,650 | 7,112 | 7,308 |  |
|  | 83 | 7,720 | 8,623 | 8,238 | 7,719 | 7,179 | 6,403 |  | 7,483 | 8,003 | 8,224 |  |
|  | 84 | 8,668 | 9,681 | 9,249 | 8,667 | 8,060 | 7,189 |  | 8,402 | 8,985 | 9,233 |  |
|  | 85 | 9,717 | 9,674 | 9,874 | 9,739 | 9,715 | 9,527 |  | 9,439 | 10,384 | 10,181 |  |
|  | 86 | 10,883 | 10,834 | 11,059 | 10,908 | 10,880 | 10,670 |  | 10,572 | 11,630 | 11,403 |  |
|  | 87 | 12,186 | 12,132 | 12,383 | 12,213 | 12,183 | 11,947 |  | 11,837 | 13,022 | 12,768 |  |
|  | 88 | 13,646 | 13,585 | 13,867 | 13,676 | 13,642 | 13,379 |  | 13,255 | 14,582 | 14,297 |  |
|  | 89 | 15,268 | 15,199 | 15,515 | 15,302 | 15,264 | 14,969 |  | 14,831 | 16,315 | 15,996 |  |
|  | 90 | 17,048 | 16,971 | 17,323 | 17,086 | 17,043 | 16,714 |  | 16,559 | 18,217 | 17,861 |  |
|  | 91 | 18,973 | 18,888 | 19,280 | 19,015 | 18,968 | 18,601 |  | 18,430 | 20,274 | 19,878 |  |
|  | 92 | 21,022 | 20,928 | 21,362 | 21,069 | 21,016 | 20,610 |  | 20,420 | 22,464 | 22,025 |  |
|  | 93 | 23,167 | 23,063 | 23,541 | 23,219 | 23,161 | 22,713 |  | 22,504 | 24,756 | 24,272 |  |
|  | 94 | 25,397 | 25,283 | 25,807 | 25,453 | 25,390 | 24,899 |  | 24,669 | 27,138 | 26,608 |  |
|  | 95 | 27,700 | 27,575 | 28,147 | 27,762 | 27,692 | 27,157 |  | 26,907 | 29,600 | 29,021 |  |
|  | 96 | 30,100 | 29,965 | 30,586 | 30,167 | 30,092 | 29,510 |  | 29,238 | 32,164 | 31,536 |  |
| ^1^Data retrieved from ABS report 3302.0 - Deaths, Australia, 2010 - TABLES 4.1-4.2.1. ^2^Quintiles defined according to the Index of Relative Socio-Economic Disadvantage of the Socio-Economic Indexes for Areas (SEIFA). ^3^Mortality rates in each remoteness or socioeconomic subgroup per sex in 5 specific age groups (i.e., 15-24y, 35-44y, 45-64y, 65-84y, and 85+) were retrieved from *Mortality inequalities in Australia 2009–2011 Table S3.1: Deaths by socioeconomic group, by sex and age group, 2009–2011*. Mortality rates for each subgroup-, sex-, and year of age-stratum was calculated by multiplying sex- and year of age-specific mortality rate in the total population with the ratio of the subgroup-specific mortality rate and the age-adjusted mortality rate of the total population. | | | | | | | | | | | | |
