# Supplementary material for: Estimated health benefits, costs, and cost-effectiveness of eliminating industrial trans-fatty acids in Australia: A modelling study
Source: PLoS Med. 2020 Nov 2;17(11):e1003407. doi: 10.1371/journal.pmed.1003407 (PMC7605626; doi:10.1371/journal.pmed.1003407)
Supplement: S4 Table — (DOCX) [file pmed.1003407.s006.docx]

**S4 Table.** Total and subgroup-specific IHD incidence (%) per year of age

|  |  |  | SEIFA Quintile^2,3^ | | | | |  | Remoteness^3^ | | |  |
| --- | --- | --- | --- | --- | --- | --- | --- | --- | --- | --- | --- | --- |
| Sex | Age (y) | Total population^1^ | 1 | 2 | 3 | 4 | 5 |  | Major cities | Inner regional | Outer regional, remote, & very remote |  |
| Women | 20 | 0.02 | 0.02 | 0.02 | 0.02 | 0.02 | 0.02 |  | 0.02 | 0.02 | 0.02 |  |
|  | 21 | 0.02 | 0.02 | 0.02 | 0.02 | 0.02 | 0.02 |  | 0.02 | 0.02 | 0.02 |  |
|  | 22 | 0.02 | 0.02 | 0.02 | 0.02 | 0.02 | 0.02 |  | 0.02 | 0.02 | 0.02 |  |
|  | 23 | 0.02 | 0.02 | 0.02 | 0.02 | 0.02 | 0.02 |  | 0.02 | 0.02 | 0.02 |  |
|  | 24 | 0.03 | 0.03 | 0.03 | 0.03 | 0.03 | 0.03 |  | 0.03 | 0.04 | 0.03 |  |
|  | 25 | 0.03 | 0.03 | 0.03 | 0.03 | 0.03 | 0.03 |  | 0.03 | 0.04 | 0.03 |  |
|  | 26 | 0.03 | 0.03 | 0.03 | 0.03 | 0.03 | 0.03 |  | 0.03 | 0.04 | 0.03 |  |
|  | 27 | 0.04 | 0.05 | 0.04 | 0.04 | 0.04 | 0.03 |  | 0.04 | 0.05 | 0.04 |  |
|  | 28 | 0.04 | 0.05 | 0.04 | 0.04 | 0.04 | 0.03 |  | 0.04 | 0.05 | 0.04 |  |
|  | 29 | 0.04 | 0.05 | 0.04 | 0.04 | 0.04 | 0.03 |  | 0.04 | 0.05 | 0.04 |  |
|  | 30 | 0.05 | 0.06 | 0.05 | 0.05 | 0.05 | 0.04 |  | 0.05 | 0.06 | 0.05 |  |
|  | 31 | 0.05 | 0.06 | 0.05 | 0.05 | 0.05 | 0.04 |  | 0.05 | 0.06 | 0.05 |  |
|  | 32 | 0.05 | 0.06 | 0.05 | 0.05 | 0.05 | 0.04 |  | 0.05 | 0.06 | 0.05 |  |
|  | 33 | 0.06 | 0.07 | 0.06 | 0.06 | 0.05 | 0.05 |  | 0.06 | 0.07 | 0.06 |  |
|  | 34 | 0.06 | 0.07 | 0.06 | 0.06 | 0.05 | 0.05 |  | 0.06 | 0.07 | 0.06 |  |
|  | 35 | 0.06 | 0.07 | 0.06 | 0.06 | 0.05 | 0.05 |  | 0.06 | 0.07 | 0.06 |  |
|  | 36 | 0.07 | 0.08 | 0.07 | 0.07 | 0.06 | 0.06 |  | 0.07 | 0.08 | 0.07 |  |
|  | 37 | 0.07 | 0.08 | 0.07 | 0.07 | 0.06 | 0.06 |  | 0.07 | 0.08 | 0.07 |  |
|  | 38 | 0.08 | 0.09 | 0.08 | 0.08 | 0.07 | 0.07 |  | 0.08 | 0.10 | 0.08 |  |
|  | 39 | 0.08 | 0.09 | 0.08 | 0.08 | 0.07 | 0.07 |  | 0.08 | 0.10 | 0.08 |  |
|  | 40 | 0.09 | 0.10 | 0.10 | 0.09 | 0.08 | 0.08 |  | 0.09 | 0.11 | 0.09 |  |
|  | 41 | 0.10 | 0.11 | 0.11 | 0.10 | 0.09 | 0.09 |  | 0.10 | 0.12 | 0.10 |  |
|  | 42 | 0.11 | 0.12 | 0.12 | 0.11 | 0.10 | 0.09 |  | 0.11 | 0.13 | 0.11 |  |
|  | 43 | 0.12 | 0.14 | 0.13 | 0.12 | 0.11 | 0.10 |  | 0.11 | 0.14 | 0.12 |  |
|  | 44 | 0.13 | 0.15 | 0.14 | 0.14 | 0.12 | 0.11 |  | 0.12 | 0.16 | 0.13 |  |
|  | 45 | 0.14 | 0.16 | 0.15 | 0.15 | 0.13 | 0.12 |  | 0.13 | 0.17 | 0.14 |  |
|  | 46 | 0.15 | 0.17 | 0.16 | 0.16 | 0.14 | 0.13 |  | 0.14 | 0.18 | 0.15 |  |
|  | 47 | 0.17 | 0.19 | 0.18 | 0.18 | 0.15 | 0.15 |  | 0.16 | 0.20 | 0.17 |  |
|  | 48 | 0.18 | 0.20 | 0.19 | 0.19 | 0.16 | 0.15 |  | 0.17 | 0.22 | 0.18 |  |
|  | 49 | 0.20 | 0.23 | 0.21 | 0.21 | 0.18 | 0.17 |  | 0.19 | 0.24 | 0.20 |  |
|  | 50 | 0.22 | 0.25 | 0.23 | 0.23 | 0.20 | 0.19 |  | 0.21 | 0.26 | 0.22 |  |
|  | 51 | 0.23 | 0.26 | 0.24 | 0.24 | 0.21 | 0.20 |  | 0.22 | 0.28 | 0.23 |  |
|  | 52 | 0.25 | 0.28 | 0.26 | 0.26 | 0.23 | 0.21 |  | 0.24 | 0.30 | 0.25 |  |
|  | 53 | 0.26 | 0.29 | 0.28 | 0.27 | 0.24 | 0.22 |  | 0.25 | 0.31 | 0.26 |  |
|  | 54 | 0.27 | 0.31 | 0.29 | 0.28 | 0.25 | 0.23 |  | 0.26 | 0.32 | 0.27 |  |
|  | 55 | 0.27 | 0.31 | 0.29 | 0.28 | 0.25 | 0.23 |  | 0.26 | 0.32 | 0.27 |  |
|  | 56 | 0.28 | 0.32 | 0.30 | 0.29 | 0.25 | 0.24 |  | 0.27 | 0.34 | 0.28 |  |
|  | 57 | 0.29 | 0.33 | 0.31 | 0.30 | 0.26 | 0.25 |  | 0.28 | 0.35 | 0.29 |  |
|  | 58 | 0.30 | 0.34 | 0.32 | 0.31 | 0.27 | 0.26 |  | 0.29 | 0.36 | 0.30 |  |
|  | 59 | 0.30 | 0.34 | 0.32 | 0.31 | 0.27 | 0.26 |  | 0.29 | 0.36 | 0.30 |  |
|  | 60 | 0.31 | 0.35 | 0.33 | 0.32 | 0.28 | 0.27 |  | 0.30 | 0.37 | 0.31 |  |
|  | 61 | 0.32 | 0.36 | 0.34 | 0.33 | 0.29 | 0.28 |  | 0.31 | 0.38 | 0.32 |  |
|  | 62 | 0.33 | 0.37 | 0.35 | 0.34 | 0.30 | 0.28 |  | 0.32 | 0.40 | 0.33 |  |
|  | 63 | 0.35 | 0.40 | 0.37 | 0.36 | 0.32 | 0.30 |  | 0.33 | 0.42 | 0.35 |  |
|  | 64 | 0.36 | 0.41 | 0.38 | 0.37 | 0.33 | 0.31 |  | 0.34 | 0.43 | 0.36 |  |
|  | 65 | 0.38 | 0.43 | 0.40 | 0.40 | 0.35 | 0.33 |  | 0.36 | 0.46 | 0.37 |  |
|  | 66 | 0.40 | 0.45 | 0.42 | 0.42 | 0.36 | 0.34 |  | 0.38 | 0.48 | 0.39 |  |
|  | 67 | 0.41 | 0.46 | 0.43 | 0.43 | 0.37 | 0.35 |  | 0.39 | 0.49 | 0.40 |  |
|  | 68 | 0.43 | 0.49 | 0.46 | 0.45 | 0.39 | 0.37 |  | 0.41 | 0.52 | 0.42 |  |
|  | 69 | 0.45 | 0.51 | 0.48 | 0.47 | 0.41 | 0.39 |  | 0.43 | 0.54 | 0.44 |  |
|  | 70 | 0.47 | 0.53 | 0.50 | 0.49 | 0.43 | 0.40 |  | 0.45 | 0.56 | 0.46 |  |
|  | 71 | 0.49 | 0.55 | 0.52 | 0.51 | 0.45 | 0.42 |  | 0.47 | 0.59 | 0.48 |  |
|  | 72 | 0.52 | 0.59 | 0.55 | 0.54 | 0.47 | 0.45 |  | 0.50 | 0.62 | 0.51 |  |
|  | 73 | 0.57 | 0.64 | 0.60 | 0.59 | 0.52 | 0.49 |  | 0.54 | 0.68 | 0.56 |  |
|  | 74 | 0.62 | 0.70 | 0.66 | 0.65 | 0.56 | 0.53 |  | 0.59 | 0.74 | 0.61 |  |
|  | 75 | 0.68 | 0.77 | 0.72 | 0.71 | 0.62 | 0.58 |  | 0.65 | 0.82 | 0.67 |  |
|  | 76 | 0.73 | 0.83 | 0.77 | 0.76 | 0.66 | 0.63 |  | 0.70 | 0.88 | 0.72 |  |
|  | 77 | 0.78 | 0.88 | 0.83 | 0.81 | 0.71 | 0.67 |  | 0.74 | 0.94 | 0.77 |  |
|  | 78 | 0.82 | 0.93 | 0.87 | 0.85 | 0.75 | 0.70 |  | 0.78 | 0.98 | 0.81 |  |
|  | 79 | 0.86 | 0.97 | 0.91 | 0.90 | 0.78 | 0.74 |  | 0.82 | 1.03 | 0.85 |  |
|  | 80 | 0.90 | 1.02 | 0.95 | 0.94 | 0.82 | 0.77 |  | 0.86 | 1.08 | 0.89 |  |
|  | 81 | 1.00 | 1.13 | 1.06 | 1.04 | 0.91 | 0.86 |  | 0.95 | 1.20 | 0.99 |  |
|  | 82 | 1.17 | 1.32 | 1.24 | 1.22 | 1.06 | 1.01 |  | 1.12 | 1.40 | 1.15 |  |
|  | 83 | 1.40 | 1.58 | 1.48 | 1.46 | 1.27 | 1.20 |  | 1.34 | 1.68 | 1.38 |  |
|  | 84 | 1.71 | 1.93 | 1.81 | 1.78 | 1.56 | 1.47 |  | 1.63 | 2.05 | 1.69 |  |
|  | 85 | 2.08 | 2.35 | 2.20 | 2.16 | 1.89 | 1.79 |  | 1.99 | 2.49 | 2.05 |  |
|  | 86 | 2.48 | 2.81 | 2.63 | 2.58 | 2.26 | 2.13 |  | 2.37 | 2.97 | 2.45 |  |
|  | 87 | 2.91 | 3.29 | 3.08 | 3.03 | 2.65 | 2.50 |  | 2.78 | 3.49 | 2.87 |  |
|  | 88 | 3.37 | 3.81 | 3.57 | 3.51 | 3.07 | 2.90 |  | 3.22 | 4.04 | 3.32 |  |
|  | 89 | 3.86 | 4.37 | 4.09 | 4.02 | 3.51 | 3.32 |  | 3.69 | 4.63 | 3.81 |  |
|  | 90 | 4.38 | 4.95 | 4.64 | 4.56 | 3.98 | 3.77 |  | 4.18 | 5.25 | 4.32 |  |
|  | 91 | 5.00 | 5.66 | 5.29 | 5.20 | 4.55 | 4.30 |  | 4.77 | 6.00 | 4.93 |  |
|  | 92 | 5.70 | 6.45 | 6.04 | 5.93 | 5.18 | 4.90 |  | 5.44 | 6.83 | 5.62 |  |
|  | 93 | 6.48 | 7.33 | 6.86 | 6.74 | 5.89 | 5.57 |  | 6.19 | 7.77 | 6.39 |  |
|  | 94 | 7.36 | 8.33 | 7.79 | 7.66 | 6.69 | 6.33 |  | 7.03 | 8.83 | 7.26 |  |
|  | 95 | 8.33 | 9.42 | 8.82 | 8.67 | 7.58 | 7.16 |  | 7.95 | 9.99 | 8.22 |  |
|  | 96 | 9.37 | 10.60 | 9.92 | 9.75 | 8.52 | 8.06 |  | 8.95 | 11.24 | 9.24 |  |
|  | 97 | 10.48 | 11.86 | 11.10 | 10.91 | 9.53 | 9.01 |  | 10.01 | 12.57 | 10.34 |  |
|  | 98 | 11.67 | 13.20 | 12.36 | 12.15 | 10.61 | 10.03 |  | 11.14 | 13.99 | 11.51 |  |
|  | 99 | 12.93 | 14.63 | 13.69 | 13.46 | 11.76 | 11.12 |  | 12.34 | 15.50 | 12.75 |  |
|  | 100 | 14.27 | 16.14 | 15.11 | 14.85 | 12.98 | 12.27 |  | 13.62 | 17.11 | 14.08 |  |
|  |  |  |  |  |  |  |  |  |  |  |  |  |
| Men | 20 | 0.02 | 0.02 | 0.02 | 0.02 | 0.02 | 0.02 |  | 0.02 | 0.02 | 0.02 |  |
|  | 21 | 0.02 | 0.02 | 0.02 | 0.02 | 0.02 | 0.02 |  | 0.02 | 0.02 | 0.02 |  |
|  | 22 | 0.03 | 0.03 | 0.03 | 0.03 | 0.03 | 0.03 |  | 0.03 | 0.03 | 0.03 |  |
|  | 23 | 0.03 | 0.03 | 0.03 | 0.03 | 0.03 | 0.03 |  | 0.03 | 0.03 | 0.03 |  |
|  | 24 | 0.04 | 0.05 | 0.04 | 0.04 | 0.04 | 0.04 |  | 0.04 | 0.04 | 0.04 |  |
|  | 25 | 0.04 | 0.05 | 0.04 | 0.04 | 0.04 | 0.04 |  | 0.04 | 0.04 | 0.04 |  |
|  | 26 | 0.04 | 0.05 | 0.04 | 0.04 | 0.04 | 0.04 |  | 0.04 | 0.04 | 0.04 |  |
|  | 27 | 0.05 | 0.06 | 0.05 | 0.05 | 0.05 | 0.05 |  | 0.05 | 0.05 | 0.05 |  |
|  | 28 | 0.05 | 0.06 | 0.05 | 0.05 | 0.05 | 0.05 |  | 0.05 | 0.05 | 0.05 |  |
|  | 29 | 0.06 | 0.07 | 0.06 | 0.06 | 0.06 | 0.06 |  | 0.06 | 0.06 | 0.06 |  |
|  | 30 | 0.06 | 0.07 | 0.06 | 0.06 | 0.06 | 0.06 |  | 0.06 | 0.06 | 0.06 |  |
|  | 31 | 0.07 | 0.08 | 0.07 | 0.07 | 0.07 | 0.07 |  | 0.07 | 0.07 | 0.07 |  |
|  | 32 | 0.07 | 0.08 | 0.07 | 0.07 | 0.07 | 0.07 |  | 0.07 | 0.07 | 0.07 |  |
|  | 33 | 0.08 | 0.09 | 0.08 | 0.08 | 0.08 | 0.08 |  | 0.08 | 0.09 | 0.08 |  |
|  | 34 | 0.08 | 0.09 | 0.08 | 0.08 | 0.08 | 0.08 |  | 0.08 | 0.09 | 0.08 |  |
|  | 35 | 0.09 | 0.10 | 0.09 | 0.09 | 0.08 | 0.09 |  | 0.09 | 0.10 | 0.09 |  |
|  | 36 | 0.09 | 0.10 | 0.09 | 0.09 | 0.08 | 0.09 |  | 0.09 | 0.10 | 0.09 |  |
|  | 37 | 0.10 | 0.11 | 0.10 | 0.10 | 0.09 | 0.10 |  | 0.10 | 0.11 | 0.10 |  |
|  | 38 | 0.11 | 0.13 | 0.11 | 0.11 | 0.10 | 0.11 |  | 0.11 | 0.12 | 0.11 |  |
|  | 39 | 0.12 | 0.14 | 0.12 | 0.12 | 0.11 | 0.12 |  | 0.12 | 0.13 | 0.12 |  |
|  | 40 | 0.13 | 0.15 | 0.13 | 0.13 | 0.12 | 0.13 |  | 0.13 | 0.14 | 0.13 |  |
|  | 41 | 0.14 | 0.16 | 0.14 | 0.14 | 0.13 | 0.14 |  | 0.14 | 0.15 | 0.14 |  |
|  | 42 | 0.16 | 0.18 | 0.15 | 0.16 | 0.15 | 0.16 |  | 0.16 | 0.17 | 0.16 |  |
|  | 43 | 0.17 | 0.19 | 0.16 | 0.17 | 0.16 | 0.17 |  | 0.17 | 0.18 | 0.17 |  |
|  | 44 | 0.19 | 0.22 | 0.18 | 0.19 | 0.18 | 0.19 |  | 0.19 | 0.20 | 0.19 |  |
|  | 45 | 0.21 | 0.24 | 0.20 | 0.21 | 0.20 | 0.21 |  | 0.21 | 0.22 | 0.21 |  |
|  | 46 | 0.23 | 0.26 | 0.22 | 0.23 | 0.22 | 0.22 |  | 0.23 | 0.25 | 0.23 |  |
|  | 47 | 0.25 | 0.29 | 0.24 | 0.25 | 0.24 | 0.24 |  | 0.25 | 0.27 | 0.25 |  |
|  | 48 | 0.28 | 0.32 | 0.27 | 0.27 | 0.26 | 0.27 |  | 0.28 | 0.30 | 0.28 |  |
|  | 49 | 0.30 | 0.34 | 0.29 | 0.29 | 0.28 | 0.29 |  | 0.30 | 0.32 | 0.30 |  |
|  | 50 | 0.33 | 0.38 | 0.32 | 0.32 | 0.31 | 0.32 |  | 0.33 | 0.35 | 0.33 |  |
|  | 51 | 0.36 | 0.41 | 0.35 | 0.35 | 0.34 | 0.35 |  | 0.36 | 0.38 | 0.36 |  |
|  | 52 | 0.38 | 0.44 | 0.37 | 0.37 | 0.36 | 0.37 |  | 0.38 | 0.41 | 0.38 |  |
|  | 53 | 0.40 | 0.46 | 0.39 | 0.39 | 0.38 | 0.39 |  | 0.40 | 0.43 | 0.40 |  |
|  | 54 | 0.42 | 0.48 | 0.41 | 0.41 | 0.40 | 0.41 |  | 0.42 | 0.45 | 0.42 |  |
|  | 55 | 0.43 | 0.49 | 0.42 | 0.42 | 0.40 | 0.42 |  | 0.43 | 0.46 | 0.43 |  |
|  | 56 | 0.45 | 0.52 | 0.44 | 0.44 | 0.42 | 0.44 |  | 0.45 | 0.48 | 0.45 |  |
|  | 57 | 0.46 | 0.53 | 0.45 | 0.45 | 0.43 | 0.45 |  | 0.46 | 0.49 | 0.46 |  |
|  | 58 | 0.48 | 0.55 | 0.46 | 0.47 | 0.45 | 0.47 |  | 0.48 | 0.51 | 0.48 |  |
|  | 59 | 0.49 | 0.56 | 0.47 | 0.48 | 0.46 | 0.48 |  | 0.49 | 0.52 | 0.49 |  |
|  | 60 | 0.50 | 0.57 | 0.48 | 0.49 | 0.47 | 0.49 |  | 0.50 | 0.53 | 0.50 |  |
|  | 61 | 0.52 | 0.60 | 0.50 | 0.51 | 0.49 | 0.51 |  | 0.52 | 0.56 | 0.52 |  |
|  | 62 | 0.55 | 0.63 | 0.53 | 0.54 | 0.52 | 0.54 |  | 0.55 | 0.59 | 0.55 |  |
|  | 63 | 0.58 | 0.66 | 0.56 | 0.57 | 0.55 | 0.57 |  | 0.57 | 0.62 | 0.58 |  |
|  | 64 | 0.61 | 0.70 | 0.59 | 0.60 | 0.57 | 0.60 |  | 0.60 | 0.65 | 0.61 |  |
|  | 65 | 0.65 | 0.74 | 0.63 | 0.64 | 0.61 | 0.64 |  | 0.64 | 0.69 | 0.65 |  |
|  | 66 | 0.68 | 0.78 | 0.66 | 0.67 | 0.64 | 0.66 |  | 0.67 | 0.73 | 0.68 |  |
|  | 67 | 0.72 | 0.82 | 0.70 | 0.71 | 0.68 | 0.70 |  | 0.71 | 0.77 | 0.72 |  |
|  | 68 | 0.75 | 0.86 | 0.73 | 0.74 | 0.71 | 0.73 |  | 0.74 | 0.80 | 0.75 |  |
|  | 69 | 0.78 | 0.89 | 0.76 | 0.77 | 0.73 | 0.76 |  | 0.77 | 0.83 | 0.78 |  |
|  | 70 | 0.80 | 0.92 | 0.77 | 0.79 | 0.75 | 0.78 |  | 0.79 | 0.85 | 0.80 |  |
|  | 71 | 0.84 | 0.96 | 0.81 | 0.82 | 0.79 | 0.82 |  | 0.83 | 0.90 | 0.84 |  |
|  | 72 | 0.90 | 1.03 | 0.87 | 0.88 | 0.85 | 0.88 |  | 0.89 | 0.96 | 0.90 |  |
|  | 73 | 0.96 | 1.10 | 0.93 | 0.94 | 0.90 | 0.94 |  | 0.95 | 1.03 | 0.96 |  |
|  | 74 | 1.05 | 1.20 | 1.02 | 1.03 | 0.99 | 1.03 |  | 1.04 | 1.12 | 1.05 |  |
|  | 75 | 1.14 | 1.31 | 1.10 | 1.12 | 1.07 | 1.11 |  | 1.13 | 1.22 | 1.14 |  |
|  | 76 | 1.24 | 1.42 | 1.20 | 1.22 | 1.17 | 1.21 |  | 1.23 | 1.32 | 1.24 |  |
|  | 77 | 1.33 | 1.52 | 1.29 | 1.31 | 1.25 | 1.30 |  | 1.32 | 1.42 | 1.33 |  |
|  | 78 | 1.42 | 1.63 | 1.37 | 1.39 | 1.34 | 1.39 |  | 1.41 | 1.52 | 1.42 |  |
|  | 79 | 1.50 | 1.72 | 1.45 | 1.47 | 1.41 | 1.47 |  | 1.49 | 1.60 | 1.50 |  |
|  | 80 | 1.59 | 1.82 | 1.54 | 1.56 | 1.50 | 1.55 |  | 1.58 | 1.70 | 1.59 |  |
|  | 81 | 1.75 | 2.00 | 1.69 | 1.72 | 1.65 | 1.71 |  | 1.73 | 1.87 | 1.75 |  |
|  | 82 | 1.98 | 2.27 | 1.92 | 1.94 | 1.86 | 1.94 |  | 1.96 | 2.12 | 1.98 |  |
|  | 83 | 2.29 | 2.62 | 2.22 | 2.25 | 2.15 | 2.24 |  | 2.27 | 2.45 | 2.29 |  |
|  | 84 | 2.68 | 3.07 | 2.59 | 2.63 | 2.52 | 2.62 |  | 2.66 | 2.86 | 2.68 |  |
|  | 85 | 3.14 | 3.60 | 3.04 | 3.08 | 2.95 | 3.07 |  | 3.11 | 3.35 | 3.14 |  |
|  | 86 | 3.63 | 4.16 | 3.51 | 3.56 | 3.42 | 3.55 |  | 3.60 | 3.88 | 3.63 |  |
|  | 87 | 4.14 | 4.74 | 4.01 | 4.06 | 3.90 | 4.05 |  | 4.10 | 4.42 | 4.14 |  |
|  | 88 | 4.67 | 5.35 | 4.52 | 4.59 | 4.39 | 4.56 |  | 4.63 | 4.99 | 4.67 |  |
|  | 89 | 5.23 | 5.99 | 5.06 | 5.13 | 4.92 | 5.11 |  | 5.18 | 5.59 | 5.23 |  |
|  | 90 | 5.81 | 6.66 | 5.63 | 5.70 | 5.47 | 5.68 |  | 5.76 | 6.21 | 5.81 |  |
|  | 91 | 6.45 | 7.39 | 6.24 | 6.33 | 6.07 | 6.30 |  | 6.39 | 6.89 | 6.45 |  |
|  | 92 | 7.14 | 8.18 | 6.91 | 7.01 | 6.72 | 6.98 |  | 7.08 | 7.63 | 7.14 |  |
|  | 93 | 7.89 | 9.04 | 7.64 | 7.75 | 7.42 | 7.71 |  | 7.82 | 8.43 | 7.89 |  |
|  | 94 | 8.70 | 9.97 | 8.42 | 8.54 | 8.19 | 8.50 |  | 8.62 | 9.29 | 8.70 |  |
|  | 95 | 9.56 | 10.95 | 9.26 | 9.39 | 9.00 | 9.34 |  | 9.47 | 10.21 | 9.56 |  |
|  | 96 | 10.17 | 11.65 | 9.85 | 9.99 | 9.57 | 9.94 |  | 10.08 | 10.86 | 10.17 |  |
|  | 97 | 10.51 | 12.04 | 10.18 | 10.32 | 9.89 | 10.27 |  | 10.41 | 11.23 | 10.51 |  |
|  | 98 | 10.60 | 12.14 | 10.26 | 10.41 | 9.97 | 10.36 |  | 10.50 | 11.32 | 10.60 |  |
|  | 99 | 10.43 | 11.95 | 10.10 | 10.24 | 9.81 | 10.19 |  | 10.34 | 11.14 | 10.43 |  |
|  | 100 | 10.00 | 11.45 | 9.68 | 9.82 | 9.41 | 9.77 |  | 9.91 | 10.68 | 10.00 |  |
| ^1^Data retrieved from Global burden of disease project 2010. ^2^Quintiles defined according to the Index of Relative Socio-Economic Disadvantage of the Socio-Economic Indexes for Areas (SEIFA). ^3^Cardivascular disease hospitalisation rates in the total population and in each remoteness or socioeconomic subgroup were retrieved from the *AIHW Cardiovascular disease web pages data tables* (https://www.aihw.gov.au/getmedia/0c4fd299-edc6-40f5-b5e1-69d48c9c9648/cvd-ccc2016-20409.xls.aspx [Accessed July 25, 2019]). IHD incidence for each subgroup-, sex-, and age-stratum was calculated by multiplying sex-age-specific IHD incidence in the total population with the ratio of the subgroup-specific CVD hospitalisation rate and the age-adjusted CVD hospitalisation rate of the total population. | | | | | | | | | | | | |
