# Supplementary material for: Estimated health benefits, costs, and cost-effectiveness of eliminating industrial trans-fatty acids in Australia: A modelling study
Source: PLoS Med. 2020 Nov 2;17(11):e1003407. doi: 10.1371/journal.pmed.1003407 (PMC7605626; doi:10.1371/journal.pmed.1003407)
Supplement: S5 Table — (DOCX) [file pmed.1003407.s007.docx]

**S5 Table.** Total and subgroup-specific IHD prevalence (%) per year of age

|  |  |  | SEIFA Quintile^2,3^ | | | | |  | Remoteness^3^ | | |  |
| --- | --- | --- | --- | --- | --- | --- | --- | --- | --- | --- | --- | --- |
| Sex | Age (y) | Total population^1^ | 1 | 2 | 3 | 4 | 5 |  | Major cities | Inner regional | Outer regional, remote, & very remote |  |
| Women | 20 | 0.09 | 0.10 | 0.10 | 0.09 | 0.08 | 0.08 |  | 0.09 | 0.11 | 0.09 |  |
|  | 21 | 0.10 | 0.11 | 0.11 | 0.10 | 0.09 | 0.09 |  | 0.10 | 0.12 | 0.10 |  |
|  | 22 | 0.12 | 0.14 | 0.13 | 0.12 | 0.11 | 0.10 |  | 0.11 | 0.14 | 0.12 |  |
|  | 23 | 0.15 | 0.17 | 0.16 | 0.16 | 0.14 | 0.13 |  | 0.14 | 0.18 | 0.15 |  |
|  | 24 | 0.17 | 0.19 | 0.18 | 0.18 | 0.15 | 0.15 |  | 0.16 | 0.20 | 0.17 |  |
|  | 25 | 0.20 | 0.23 | 0.21 | 0.21 | 0.18 | 0.17 |  | 0.19 | 0.24 | 0.20 |  |
|  | 26 | 0.23 | 0.26 | 0.24 | 0.24 | 0.21 | 0.20 |  | 0.22 | 0.28 | 0.23 |  |
|  | 27 | 0.26 | 0.29 | 0.28 | 0.27 | 0.24 | 0.22 |  | 0.25 | 0.31 | 0.26 |  |
|  | 28 | 0.30 | 0.34 | 0.32 | 0.31 | 0.27 | 0.26 |  | 0.29 | 0.36 | 0.30 |  |
|  | 29 | 0.33 | 0.37 | 0.35 | 0.34 | 0.30 | 0.28 |  | 0.32 | 0.40 | 0.33 |  |
|  | 30 | 0.38 | 0.43 | 0.40 | 0.40 | 0.35 | 0.33 |  | 0.36 | 0.46 | 0.37 |  |
|  | 31 | 0.42 | 0.48 | 0.44 | 0.44 | 0.38 | 0.36 |  | 0.40 | 0.50 | 0.41 |  |
|  | 32 | 0.47 | 0.53 | 0.50 | 0.49 | 0.43 | 0.40 |  | 0.45 | 0.56 | 0.46 |  |
|  | 33 | 0.53 | 0.60 | 0.56 | 0.55 | 0.48 | 0.46 |  | 0.51 | 0.64 | 0.52 |  |
|  | 34 | 0.58 | 0.66 | 0.61 | 0.60 | 0.53 | 0.50 |  | 0.55 | 0.70 | 0.57 |  |
|  | 35 | 0.64 | 0.72 | 0.68 | 0.67 | 0.58 | 0.55 |  | 0.61 | 0.77 | 0.63 |  |
|  | 36 | 0.70 | 0.79 | 0.74 | 0.73 | 0.64 | 0.60 |  | 0.67 | 0.84 | 0.69 |  |
|  | 37 | 0.77 | 0.87 | 0.82 | 0.80 | 0.70 | 0.66 |  | 0.74 | 0.92 | 0.76 |  |
|  | 38 | 0.84 | 0.95 | 0.89 | 0.87 | 0.76 | 0.72 |  | 0.80 | 1.01 | 0.83 |  |
|  | 39 | 0.91 | 1.03 | 0.96 | 0.95 | 0.83 | 0.78 |  | 0.87 | 1.09 | 0.90 |  |
|  | 40 | 1.00 | 1.13 | 1.06 | 1.04 | 0.91 | 0.86 |  | 0.95 | 1.20 | 0.99 |  |
|  | 41 | 1.09 | 1.23 | 1.15 | 1.13 | 0.99 | 0.94 |  | 1.04 | 1.31 | 1.08 |  |
|  | 42 | 1.19 | 1.35 | 1.26 | 1.24 | 1.08 | 1.02 |  | 1.14 | 1.43 | 1.17 |  |
|  | 43 | 1.29 | 1.46 | 1.37 | 1.34 | 1.17 | 1.11 |  | 1.23 | 1.55 | 1.27 |  |
|  | 44 | 1.41 | 1.60 | 1.49 | 1.47 | 1.28 | 1.21 |  | 1.35 | 1.69 | 1.39 |  |
|  | 45 | 1.54 | 1.74 | 1.63 | 1.60 | 1.40 | 1.32 |  | 1.47 | 1.85 | 1.52 |  |
|  | 46 | 1.68 | 1.90 | 1.78 | 1.75 | 1.53 | 1.44 |  | 1.60 | 2.01 | 1.66 |  |
|  | 47 | 1.83 | 2.07 | 1.94 | 1.90 | 1.66 | 1.57 |  | 1.75 | 2.19 | 1.81 |  |
|  | 48 | 1.99 | 2.25 | 2.11 | 2.07 | 1.81 | 1.71 |  | 1.90 | 2.39 | 1.96 |  |
|  | 49 | 2.17 | 2.45 | 2.30 | 2.26 | 1.97 | 1.87 |  | 2.07 | 2.60 | 2.14 |  |
|  | 50 | 2.36 | 2.67 | 2.50 | 2.46 | 2.15 | 2.03 |  | 2.25 | 2.83 | 2.33 |  |
|  | 51 | 2.57 | 2.91 | 2.72 | 2.67 | 2.34 | 2.21 |  | 2.45 | 3.08 | 2.54 |  |
|  | 52 | 2.80 | 3.17 | 2.96 | 2.91 | 2.55 | 2.41 |  | 2.67 | 3.36 | 2.76 |  |
|  | 53 | 3.03 | 3.43 | 3.21 | 3.15 | 2.76 | 2.60 |  | 2.89 | 3.63 | 2.99 |  |
|  | 54 | 3.27 | 3.70 | 3.46 | 3.40 | 2.97 | 2.81 |  | 3.12 | 3.92 | 3.23 |  |
|  | 55 | 3.51 | 3.97 | 3.72 | 3.65 | 3.19 | 3.02 |  | 3.35 | 4.21 | 3.46 |  |
|  | 56 | 3.76 | 4.25 | 3.98 | 3.91 | 3.42 | 3.23 |  | 3.59 | 4.51 | 3.71 |  |
|  | 57 | 4.01 | 4.54 | 4.25 | 4.17 | 3.65 | 3.45 |  | 3.83 | 4.81 | 3.96 |  |
|  | 58 | 4.26 | 4.82 | 4.51 | 4.43 | 3.87 | 3.66 |  | 4.07 | 5.11 | 4.20 |  |
|  | 59 | 4.52 | 5.11 | 4.79 | 4.70 | 4.11 | 3.89 |  | 4.32 | 5.42 | 4.46 |  |
|  | 60 | 4.78 | 5.41 | 5.06 | 4.97 | 4.35 | 4.11 |  | 4.56 | 5.73 | 4.72 |  |
|  | 61 | 5.04 | 5.70 | 5.34 | 5.25 | 4.58 | 4.33 |  | 4.81 | 6.04 | 4.97 |  |
|  | 62 | 5.31 | 6.01 | 5.62 | 5.53 | 4.83 | 4.57 |  | 5.07 | 6.37 | 5.24 |  |
|  | 63 | 5.58 | 6.31 | 5.91 | 5.81 | 5.08 | 4.80 |  | 5.33 | 6.69 | 5.50 |  |
|  | 64 | 5.86 | 6.63 | 6.20 | 6.10 | 5.33 | 5.04 |  | 5.59 | 7.03 | 5.78 |  |
|  | 65 | 6.14 | 6.95 | 6.50 | 6.39 | 5.58 | 5.28 |  | 5.86 | 7.36 | 6.06 |  |
|  | 66 | 6.43 | 7.27 | 6.81 | 6.69 | 5.85 | 5.53 |  | 6.14 | 7.71 | 6.34 |  |
|  | 67 | 6.72 | 7.60 | 7.12 | 6.99 | 6.11 | 5.78 |  | 6.42 | 8.06 | 6.63 |  |
|  | 68 | 7.01 | 7.93 | 7.42 | 7.30 | 6.38 | 6.03 |  | 6.69 | 8.41 | 6.91 |  |
|  | 69 | 7.30 | 8.26 | 7.73 | 7.60 | 6.64 | 6.28 |  | 6.97 | 8.75 | 7.20 |  |
|  | 70 | 7.58 | 8.57 | 8.03 | 7.89 | 6.89 | 6.52 |  | 7.24 | 9.09 | 7.48 |  |
|  | 71 | 7.86 | 8.89 | 8.32 | 8.18 | 7.15 | 6.76 |  | 7.50 | 9.42 | 7.75 |  |
|  | 72 | 8.14 | 9.21 | 8.62 | 8.47 | 7.40 | 7.00 |  | 7.77 | 9.76 | 8.03 |  |
|  | 73 | 8.42 | 9.52 | 8.92 | 8.76 | 7.66 | 7.24 |  | 8.04 | 10.10 | 8.31 |  |
|  | 74 | 8.70 | 9.84 | 9.21 | 9.05 | 7.91 | 7.48 |  | 8.31 | 10.43 | 8.58 |  |
|  | 75 | 8.98 | 10.16 | 9.51 | 9.35 | 8.17 | 7.72 |  | 8.57 | 10.77 | 8.86 |  |
|  | 76 | 9.26 | 10.48 | 9.80 | 9.64 | 8.42 | 7.96 |  | 8.84 | 11.10 | 9.13 |  |
|  | 77 | 9.52 | 10.77 | 10.08 | 9.91 | 8.66 | 8.18 |  | 9.09 | 11.42 | 9.39 |  |
|  | 78 | 9.74 | 11.02 | 10.31 | 10.14 | 8.86 | 8.37 |  | 9.30 | 11.68 | 9.61 |  |
|  | 79 | 9.91 | 11.21 | 10.49 | 10.31 | 9.01 | 8.52 |  | 9.46 | 11.88 | 9.78 |  |
|  | 80 | 10.02 | 11.33 | 10.61 | 10.43 | 9.11 | 8.61 |  | 9.57 | 12.01 | 9.88 |  |
|  | 81 | 10.07 | 11.39 | 10.66 | 10.48 | 9.16 | 8.66 |  | 9.61 | 12.07 | 9.93 |  |
|  | 82 | 10.12 | 11.45 | 10.72 | 10.53 | 9.20 | 8.70 |  | 9.66 | 12.13 | 9.98 |  |
|  | 83 | 10.21 | 11.55 | 10.81 | 10.63 | 9.29 | 8.78 |  | 9.75 | 12.24 | 10.07 |  |
|  | 84 | 10.37 | 11.73 | 10.98 | 10.79 | 9.43 | 8.92 |  | 9.90 | 12.43 | 10.23 |  |
|  | 85 | 10.63 | 12.02 | 11.26 | 11.06 | 9.67 | 9.14 |  | 10.15 | 12.75 | 10.49 |  |
|  | 86 | 11.00 | 12.44 | 11.65 | 11.45 | 10.00 | 9.46 |  | 10.50 | 13.19 | 10.85 |  |
|  | 87 | 11.44 | 12.94 | 12.11 | 11.91 | 10.40 | 9.84 |  | 10.92 | 13.72 | 11.28 |  |
|  | 88 | 11.93 | 13.50 | 12.63 | 12.42 | 10.85 | 10.26 |  | 11.39 | 14.31 | 11.77 |  |
|  | 89 | 12.45 | 14.08 | 13.18 | 12.96 | 11.32 | 10.70 |  | 11.89 | 14.93 | 12.28 |  |
|  | 90 | 12.98 | 14.68 | 13.74 | 13.51 | 11.81 | 11.16 |  | 12.39 | 15.56 | 12.80 |  |
|  | 91 | 13.49 | 15.26 | 14.28 | 14.04 | 12.27 | 11.60 |  | 12.88 | 16.18 | 13.31 |  |
|  | 92 | 14.01 | 15.85 | 14.83 | 14.58 | 12.74 | 12.04 |  | 13.38 | 16.80 | 13.82 |  |
|  | 93 | 14.52 | 16.43 | 15.37 | 15.11 | 13.21 | 12.48 |  | 13.86 | 17.41 | 14.32 |  |
|  | 94 | 15.03 | 17.00 | 15.91 | 15.64 | 13.67 | 12.92 |  | 14.35 | 18.02 | 14.83 |  |
|  | 95 | 15.54 | 17.58 | 16.45 | 16.17 | 14.13 | 13.36 |  | 14.84 | 18.63 | 15.33 |  |
|  | 96 | 15.88 | 17.96 | 16.81 | 16.53 | 14.44 | 13.65 |  | 15.16 | 19.04 | 15.66 |  |
|  | 97 | 15.84 | 17.92 | 16.77 | 16.49 | 14.41 | 13.62 |  | 15.12 | 18.99 | 15.62 |  |
|  | 98 | 15.31 | 17.32 | 16.21 | 15.93 | 13.92 | 13.16 |  | 14.62 | 18.36 | 15.10 |  |
|  | 99 | 14.38 | 16.27 | 15.23 | 14.97 | 13.08 | 12.36 |  | 13.73 | 17.24 | 14.18 |  |
|  | 100 | 13.22 | 14.95 | 14.00 | 13.76 | 12.02 | 11.37 |  | 12.62 | 15.85 | 13.04 |  |
|  |  |  |  |  |  |  |  |  |  |  |  |  |
| Men | 20 | 0.12 | 0.14 | 0.12 | 0.12 | 0.11 | 0.12 |  | 0.12 | 0.13 | 0.12 |  |
|  | 21 | 0.14 | 0.16 | 0.14 | 0.14 | 0.13 | 0.14 |  | 0.14 | 0.15 | 0.14 |  |
|  | 22 | 0.17 | 0.19 | 0.16 | 0.17 | 0.16 | 0.17 |  | 0.17 | 0.18 | 0.17 |  |
|  | 23 | 0.19 | 0.22 | 0.18 | 0.19 | 0.18 | 0.19 |  | 0.19 | 0.20 | 0.19 |  |
|  | 24 | 0.23 | 0.26 | 0.22 | 0.23 | 0.22 | 0.22 |  | 0.23 | 0.25 | 0.23 |  |
|  | 25 | 0.26 | 0.30 | 0.25 | 0.26 | 0.24 | 0.25 |  | 0.26 | 0.28 | 0.26 |  |
|  | 26 | 0.31 | 0.36 | 0.30 | 0.30 | 0.29 | 0.30 |  | 0.31 | 0.33 | 0.31 |  |
|  | 27 | 0.35 | 0.40 | 0.34 | 0.34 | 0.33 | 0.34 |  | 0.35 | 0.37 | 0.35 |  |
|  | 28 | 0.40 | 0.46 | 0.39 | 0.39 | 0.38 | 0.39 |  | 0.40 | 0.43 | 0.40 |  |
|  | 29 | 0.46 | 0.53 | 0.45 | 0.45 | 0.43 | 0.45 |  | 0.46 | 0.49 | 0.46 |  |
|  | 30 | 0.51 | 0.58 | 0.49 | 0.50 | 0.48 | 0.50 |  | 0.51 | 0.54 | 0.51 |  |
|  | 31 | 0.58 | 0.66 | 0.56 | 0.57 | 0.55 | 0.57 |  | 0.57 | 0.62 | 0.58 |  |
|  | 32 | 0.64 | 0.73 | 0.62 | 0.63 | 0.60 | 0.63 |  | 0.63 | 0.68 | 0.64 |  |
|  | 33 | 0.71 | 0.81 | 0.69 | 0.70 | 0.67 | 0.69 |  | 0.70 | 0.76 | 0.71 |  |
|  | 34 | 0.78 | 0.89 | 0.76 | 0.77 | 0.73 | 0.76 |  | 0.77 | 0.83 | 0.78 |  |
|  | 35 | 0.86 | 0.99 | 0.83 | 0.84 | 0.81 | 0.84 |  | 0.85 | 0.92 | 0.86 |  |
|  | 36 | 0.94 | 1.08 | 0.91 | 0.92 | 0.88 | 0.92 |  | 0.93 | 1.00 | 0.94 |  |
|  | 37 | 1.03 | 1.18 | 1.00 | 1.01 | 0.97 | 1.01 |  | 1.02 | 1.10 | 1.03 |  |
|  | 38 | 1.12 | 1.28 | 1.08 | 1.10 | 1.05 | 1.09 |  | 1.11 | 1.20 | 1.12 |  |
|  | 39 | 1.22 | 1.40 | 1.18 | 1.20 | 1.15 | 1.19 |  | 1.21 | 1.30 | 1.22 |  |
|  | 40 | 1.33 | 1.52 | 1.29 | 1.31 | 1.25 | 1.30 |  | 1.32 | 1.42 | 1.33 |  |
|  | 41 | 1.45 | 1.66 | 1.40 | 1.42 | 1.36 | 1.42 |  | 1.44 | 1.55 | 1.45 |  |
|  | 42 | 1.58 | 1.81 | 1.53 | 1.55 | 1.49 | 1.54 |  | 1.57 | 1.69 | 1.58 |  |
|  | 43 | 1.73 | 1.98 | 1.67 | 1.70 | 1.63 | 1.69 |  | 1.71 | 1.85 | 1.73 |  |
|  | 44 | 1.88 | 2.15 | 1.82 | 1.85 | 1.77 | 1.84 |  | 1.86 | 2.01 | 1.88 |  |
|  | 45 | 2.05 | 2.35 | 1.98 | 2.01 | 1.93 | 2.00 |  | 2.03 | 2.19 | 2.05 |  |
|  | 46 | 2.24 | 2.57 | 2.17 | 2.20 | 2.11 | 2.19 |  | 2.22 | 2.39 | 2.24 |  |
|  | 47 | 2.44 | 2.79 | 2.36 | 2.40 | 2.30 | 2.38 |  | 2.42 | 2.61 | 2.44 |  |
|  | 48 | 2.66 | 3.05 | 2.58 | 2.61 | 2.50 | 2.60 |  | 2.64 | 2.84 | 2.66 |  |
|  | 49 | 2.90 | 3.32 | 2.81 | 2.85 | 2.73 | 2.83 |  | 2.87 | 3.10 | 2.90 |  |
|  | 50 | 3.16 | 3.62 | 3.06 | 3.10 | 2.97 | 3.09 |  | 3.13 | 3.38 | 3.16 |  |
|  | 51 | 3.43 | 3.93 | 3.32 | 3.37 | 3.23 | 3.35 |  | 3.40 | 3.66 | 3.43 |  |
|  | 52 | 3.73 | 4.27 | 3.61 | 3.66 | 3.51 | 3.65 |  | 3.70 | 3.98 | 3.73 |  |
|  | 53 | 4.04 | 4.63 | 3.91 | 3.97 | 3.80 | 3.95 |  | 4.00 | 4.32 | 4.04 |  |
|  | 54 | 4.36 | 4.99 | 4.22 | 4.28 | 4.10 | 4.26 |  | 4.32 | 4.66 | 4.36 |  |
|  | 55 | 4.69 | 5.37 | 4.54 | 4.60 | 4.41 | 4.58 |  | 4.65 | 5.01 | 4.69 |  |
|  | 56 | 5.03 | 5.76 | 4.87 | 4.94 | 4.73 | 4.92 |  | 4.98 | 5.37 | 5.03 |  |
|  | 57 | 5.37 | 6.15 | 5.20 | 5.27 | 5.05 | 5.25 |  | 5.32 | 5.74 | 5.37 |  |
|  | 58 | 5.71 | 6.54 | 5.53 | 5.61 | 5.37 | 5.58 |  | 5.66 | 6.10 | 5.71 |  |
|  | 59 | 6.06 | 6.94 | 5.87 | 5.95 | 5.70 | 5.92 |  | 6.00 | 6.47 | 6.06 |  |
|  | 60 | 6.41 | 7.34 | 6.21 | 6.29 | 6.03 | 6.26 |  | 6.35 | 6.85 | 6.41 |  |
|  | 61 | 6.76 | 7.74 | 6.54 | 6.64 | 6.36 | 6.61 |  | 6.70 | 7.22 | 6.76 |  |
|  | 62 | 7.12 | 8.16 | 6.89 | 6.99 | 6.70 | 6.96 |  | 7.06 | 7.61 | 7.12 |  |
|  | 63 | 7.49 | 8.58 | 7.25 | 7.35 | 7.05 | 7.32 |  | 7.42 | 8.00 | 7.49 |  |
|  | 64 | 7.86 | 9.00 | 7.61 | 7.72 | 7.40 | 7.68 |  | 7.79 | 8.40 | 7.86 |  |
|  | 65 | 8.25 | 9.45 | 7.99 | 8.10 | 7.76 | 8.06 |  | 8.18 | 8.81 | 8.25 |  |
|  | 66 | 8.65 | 9.91 | 8.37 | 8.49 | 8.14 | 8.45 |  | 8.57 | 9.24 | 8.65 |  |
|  | 67 | 9.05 | 10.37 | 8.76 | 8.89 | 8.52 | 8.84 |  | 8.97 | 9.67 | 9.05 |  |
|  | 68 | 9.46 | 10.84 | 9.16 | 9.29 | 8.90 | 9.25 |  | 9.37 | 10.11 | 9.46 |  |
|  | 69 | 9.87 | 11.31 | 9.56 | 9.69 | 9.29 | 9.65 |  | 9.78 | 10.54 | 9.87 |  |
|  | 70 | 10.26 | 11.75 | 9.93 | 10.07 | 9.65 | 10.03 |  | 10.17 | 10.96 | 10.26 |  |
|  | 71 | 10.65 | 12.20 | 10.31 | 10.46 | 10.02 | 10.41 |  | 10.55 | 11.38 | 10.65 |  |
|  | 72 | 11.04 | 12.65 | 10.69 | 10.84 | 10.39 | 10.79 |  | 10.94 | 11.79 | 11.04 |  |
|  | 73 | 11.44 | 13.10 | 11.08 | 11.23 | 10.76 | 11.18 |  | 11.34 | 12.22 | 11.44 |  |
|  | 74 | 11.84 | 13.56 | 11.46 | 11.62 | 11.14 | 11.57 |  | 11.73 | 12.65 | 11.84 |  |
|  | 75 | 12.25 | 14.03 | 11.86 | 12.03 | 11.53 | 11.97 |  | 12.14 | 13.09 | 12.25 |  |
|  | 76 | 12.66 | 14.50 | 12.26 | 12.43 | 11.91 | 12.37 |  | 12.54 | 13.52 | 12.66 |  |
|  | 77 | 13.06 | 14.96 | 12.64 | 12.82 | 12.29 | 12.76 |  | 12.94 | 13.95 | 13.06 |  |
|  | 78 | 13.41 | 15.36 | 12.98 | 13.17 | 12.62 | 13.11 |  | 13.29 | 14.32 | 13.41 |  |
|  | 79 | 13.72 | 15.72 | 13.28 | 13.47 | 12.91 | 13.41 |  | 13.60 | 14.66 | 13.72 |  |
|  | 80 | 13.96 | 15.99 | 13.52 | 13.71 | 13.14 | 13.64 |  | 13.83 | 14.91 | 13.96 |  |
|  | 81 | 14.15 | 16.21 | 13.70 | 13.89 | 13.31 | 13.83 |  | 14.02 | 15.11 | 14.15 |  |
|  | 82 | 14.35 | 16.44 | 13.89 | 14.09 | 13.50 | 14.02 |  | 14.22 | 15.33 | 14.35 |  |
|  | 83 | 14.60 | 16.72 | 14.14 | 14.33 | 13.74 | 14.27 |  | 14.47 | 15.60 | 14.60 |  |
|  | 84 | 14.93 | 17.10 | 14.45 | 14.66 | 14.05 | 14.59 |  | 14.79 | 15.95 | 14.93 |  |
|  | 85 | 15.39 | 17.63 | 14.90 | 15.11 | 14.48 | 15.04 |  | 15.25 | 16.44 | 15.39 |  |
|  | 86 | 15.96 | 18.28 | 15.45 | 15.67 | 15.02 | 15.60 |  | 15.81 | 17.05 | 15.96 |  |
|  | 87 | 16.63 | 19.05 | 16.10 | 16.33 | 15.65 | 16.25 |  | 16.48 | 17.76 | 16.63 |  |
|  | 88 | 17.37 | 19.90 | 16.82 | 17.05 | 16.34 | 16.98 |  | 17.21 | 18.55 | 17.37 |  |
|  | 89 | 18.14 | 20.78 | 17.56 | 17.81 | 17.07 | 17.73 |  | 17.98 | 19.38 | 18.14 |  |
|  | 90 | 18.92 | 21.67 | 18.32 | 18.58 | 17.80 | 18.49 |  | 18.75 | 20.21 | 18.92 |  |
|  | 91 | 19.70 | 22.57 | 19.07 | 19.34 | 18.54 | 19.25 |  | 19.52 | 21.04 | 19.70 |  |
|  | 92 | 20.47 | 23.45 | 19.82 | 20.10 | 19.26 | 20.00 |  | 20.28 | 21.87 | 20.47 |  |
|  | 93 | 21.23 | 24.32 | 20.55 | 20.84 | 19.98 | 20.75 |  | 21.04 | 22.68 | 21.23 |  |
|  | 94 | 21.96 | 25.15 | 21.26 | 21.56 | 20.66 | 21.46 |  | 21.76 | 23.46 | 21.96 |  |
|  | 95 | 22.66 | 25.96 | 21.94 | 22.25 | 21.32 | 22.15 |  | 22.45 | 24.21 | 22.66 |  |
|  | 96 | 23.08 | 26.44 | 22.35 | 22.66 | 21.72 | 22.56 |  | 22.87 | 24.65 | 23.08 |  |
|  | 97 | 22.81 | 26.13 | 22.08 | 22.40 | 21.46 | 22.29 |  | 22.60 | 24.37 | 22.81 |  |
|  | 98 | 21.61 | 24.75 | 20.92 | 21.22 | 20.33 | 21.12 |  | 21.41 | 23.08 | 21.61 |  |
|  | 99 | 19.47 | 22.30 | 18.85 | 19.12 | 18.32 | 19.03 |  | 19.29 | 20.80 | 19.47 |  |
|  | 100 | 16.60 | 19.01 | 16.07 | 16.30 | 15.62 | 16.22 |  | 16.45 | 17.73 | 16.60 |  |
| ^1^Data retrieved from Global burden of disease project 2010. ^2^Quintiles defined according to the Index of Relative Socio-Economic Disadvantage of the Socio-Economic Indexes for Areas (SEIFA). ^3^Cardivascular disease hospitalisation rates in the total population and in each remoteness or socioeconomic subgroup were retrieved from the *AIHW Cardiovascular disease web pages data tables* (https://www.aihw.gov.au/getmedia/0c4fd299-edc6-40f5-b5e1-69d48c9c9648/cvd-ccc2016-20409.xls.aspx [Accessed July 25, 2019]). IHD prevalence for each subgroup-, sex-, and age-stratum was calculated by multiplying sex-age-specific IHD incidence in the total population with the ratio of the subgroup-specific CVD hospitalisation rate and the age-adjusted CVD hospitalisation rate of the total population. | | | | | | | | | | | | |
