# Supplementary material for: Estimated health benefits, costs, and cost-effectiveness of eliminating industrial trans-fatty acids in Australia: A modelling study
Source: PLoS Med. 2020 Nov 2;17(11):e1003407. doi: 10.1371/journal.pmed.1003407 (PMC7605626; doi:10.1371/journal.pmed.1003407)
Supplement: S6 Table — (DOCX) [file pmed.1003407.s008.docx]

**S6 Table.** IHD case fatality (%) per sex and year of age.^1^

| Age (y) | Women | Men |
| --- | --- | --- |
| 20 | 0.20 | 0.42 |
| 21 | 0.21 | 0.41 |
| 22 | 0.22 | 0.41 |
| 23 | 0.22 | 0.41 |
| 24 | 0.23 | 0.40 |
| 25 | 0.23 | 0.40 |
| 26 | 0.23 | 0.41 |
| 27 | 0.23 | 0.43 |
| 28 | 0.23 | 0.45 |
| 29 | 0.23 | 0.48 |
| 30 | 0.23 | 0.51 |
| 31 | 0.23 | 0.56 |
| 32 | 0.24 | 0.61 |
| 33 | 0.25 | 0.67 |
| 34 | 0.25 | 0.74 |
| 35 | 0.26 | 0.81 |
| 36 | 0.27 | 0.88 |
| 37 | 0.29 | 0.95 |
| 38 | 0.30 | 1.01 |
| 39 | 0.32 | 1.06 |
| 40 | 0.34 | 1.11 |
| 41 | 0.36 | 1.17 |
| 42 | 0.37 | 1.23 |
| 43 | 0.39 | 1.29 |
| 44 | 0.40 | 1.36 |
| 45 | 0.41 | 1.44 |
| 46 | 0.42 | 1.50 |
| 47 | 0.43 | 1.55 |
| 48 | 0.44 | 1.59 |
| 49 | 0.45 | 1.62 |
| 50 | 0.46 | 1.64 |
| 51 | 0.47 | 1.66 |
| 52 | 0.49 | 1.68 |
| 53 | 0.50 | 1.70 |
| 54 | 0.52 | 1.72 |
| 55 | 0.54 | 1.74 |
| 56 | 0.57 | 1.77 |
| 57 | 0.61 | 1.81 |
| 58 | 0.66 | 1.87 |
| 59 | 0.72 | 1.94 |
| 60 | 0.79 | 2.03 |
| 61 | 0.87 | 2.13 |
| 62 | 0.96 | 2.24 |
| 63 | 1.05 | 2.36 |
| 64 | 1.14 | 2.49 |
| 65 | 1.24 | 2.64 |
| 66 | 1.37 | 2.80 |
| 67 | 1.52 | 2.97 |
| 68 | 1.70 | 3.15 |
| 69 | 1.90 | 3.34 |
| 70 | 2.13 | 3.54 |
| 71 | 2.41 | 3.80 |
| 72 | 2.74 | 4.13 |
| 73 | 3.12 | 4.51 |
| 74 | 3.55 | 4.96 |
| 75 | 4.02 | 5.47 |
| 76 | 4.62 | 6.09 |
| 77 | 5.34 | 6.82 |
| 78 | 6.18 | 7.67 |
| 79 | 7.14 | 8.62 |
| 80 | 8.22 | 9.69 |
| 81 | 9.47 | 10.85 |
| 82 | 10.89 | 12.10 |
| 83 | 12.48 | 13.44 |
| 84 | 14.25 | 14.87 |
| 85 | 16.18 | 16.39 |
| 86 | 18.34 | 18.01 |
| 87 | 20.72 | 19.74 |
| 88 | 23.32 | 21.58 |
| 89 | 26.14 | 23.51 |
| 90 | 29.18 | 25.55 |
| 91 | 32.58 | 27.76 |
| 92 | 36.33 | 30.13 |
| 93 | 40.43 | 32.66 |
| 94 | 44.88 | 35.36 |
| 95 | 49.68 | 38.23 |
| 96 | 57.57 | 43.23 |
| 97 | 68.55 | 50.36 |
| 98 | 82.60 | 59.62 |
| 99 | 99.75 | 71.01 |

^1^Data retrieved from the Global burden of disease project 2010
