# Supplementary material for: Estimated health benefits, costs, and cost-effectiveness of eliminating industrial trans-fatty acids in Australia: A modelling study
Source: PLoS Med. 2020 Nov 2;17(11):e1003407. doi: 10.1371/journal.pmed.1003407 (PMC7605626; doi:10.1371/journal.pmed.1003407)
Supplement: S7 Table — (DOCX) [file pmed.1003407.s009.docx]

**S7 Table.** Cost-effectiveness of eliminating industrial trans-fatty acids in Australia under alternating assumptions regarding distribution of post-ban trans-fatty acid intake.^1^

| Post-ban trans-fatty acid intake^2^ | | | |  | First 10 years ICER (95% UI)^3^ | |  | Population life time ICER (95% UI)^2^ | |
| --- | --- | --- | --- | --- | --- | --- | --- | --- | --- |
| Mean (%E) | SD (%E) | 90^th^ percentile | 95^th^ percentile |  | $/HALY | % of threshold^4^ |  | $/HALY | % of threshold^3^ |
| 0.50 | 0.050^5^ | 0.57 | 0.59 |  | 1,073 (-2,257; 3,503) | 0.63 (-1.33; 2.07) |  | 1,956 (1,010; 2,750) | 1.15 (0.60; 1.62) |
|  | 0.250^6^ | 0.82 | 0.97 |  | 5,578 (946; 7,805) | 3.29 (0.56; 4.61) |  | 2,943 (1,701; 3,742) | 1.74 (1.00; 2.21) |
| 0.52 | 0.052^5^ | 0.59 | 0.61 |  | 1,616 (-1,711; 3,919) | 0.95 (-1.01; 2.31) |  | 2,059 (1,101; 2,795) | 1.22 (0.65; 1.65) |
|  | 0.260^6^ | 0.85 | 1.01 |  | 7,060 (1,750; 9,421) | 4.17 (1.03; 5.56) |  | 3,273 (1,874; 4,102) | 1.93 (1.11; 2.42) |
| 0.54 | 0.054^5^ | 0.61 | 0.63 |  | 2,291 (-1,635; 4,853) | 1.35 (-0.97; 2.71) |  | 2,207 (1,136; 2,977) | 1.30 (0.67; 1.76) |
|  | 0.270^6^ | 0.88 | 1.05 |  | 9,399 (3,198; 11,473) | 5.55 (1.89; 6.77) |  | 3,841 (2,216; 4,587) | 2.27 (1.31; 2.71) |
| 0.56 | 0.056^5^ | 0.63 | 0.66 |  | 3,115 (-877; 5,419) | 1.84 (-0.52; 3.20) |  | 2,387 (1,321; 3,145) | 1.41 (0.78; 1.86) |
|  | 0.280^6^ | 0.92 | 1.09 |  | 12,573 (4,719; 14,840) | 7.42 (2.79; 8.76) |  | 4,632 (2,541; 5,341) | 2.73 (1.50; 3.15) |
| 0.58 | 0.058^5^ | 0.66 | 0.68 |  | 4,341 (209; 6,684) | 2.56 (0.12; 3.95) |  | 2,642 (1,556; 3,473) | 1.56 (0.92; 2.05) |
|  | 0.290^6^ | 0.95 | 1.13 |  | 18,187 (7,811; 21,324) | 10.74 (4.61; 12.59) |  | 6,114 (3,381; 6,847) | 3.61 (2.00; 4.04) |
| 0.60 | 0.060^5^ | 0.68 | 0.70 |  | 6,230 (1,009; 8,408) | 3.68 (0.60; 4.96) |  | 3,046 (1,635; 3,850) | 1.80 (0.97; 2.27) |
|  | 0.300^6^ | 0.98 | 1.17 |  | 26,465 (11,386; 33,119) | 15.63 (6.72; 19.56) |  | 8,308 (4,090; 9,352) | 4.91 (2.42; 5.52) |

1Post-ban trans-fatty acid intake were assumed equal in all age-and-sex-strata. We assumed that the ban would not increase coronary heart disease risk and thus, potential impact fractions <0 were imputed as 0.

^2^Pre- and post-ban trans-fatty acid intakes were assumed to fit a lognormal distribution.

^3^Point estimates and 95% uncertainty intervals (UI) were represented by the median and 2.5^th^ and 97.5^th^ percentiles of n=1,000 Monte Carlo simulations.

^4^The ICER was expressed as Australian $ per HALY gained or as a percentage of the cost-effectiveness threshold defined as the value of a statistical life year in 2007 inflated to 2010 using consumer price index.

^5^SD assumed to 10% of mean.

^6^SD assumed to 50% of mean.
