# Supplementary material for: Estimated health benefits, costs, and cost-effectiveness of eliminating industrial trans-fatty acids in Australia: A modelling study
Source: PLoS Med. 2020 Nov 2;17(11):e1003407. doi: 10.1371/journal.pmed.1003407 (PMC7605626; doi:10.1371/journal.pmed.1003407)
Supplement: Attachment — Submitted filename: Responses to editors and reviewers - third draft.docx [file pmed.1003407.s010.docx]

Table 1. Requests from the editors

| **Comment** | **Response** | **Modifications made** |
| --- | --- | --- |
| We'd suggest revising the title according to PLOS Medicine's style - would recommend adding a subtitle after the study question, summarising the study design (eg :modelling study). | We have added a subtitle as suggested. | Title:  Estimated health benefits, costs, and cost-effectiveness of eliminating industrial trans-fatty acids in Australia: modelling study |
| We'd recommend noting any key limitations of the study methodology in the abstract, for example stating here that the TFA intake data date back to 2011/2012 and therefore more recent changes in intake may affect interpretability of these findings. | We have added key limitations to the abstract. | Abstract:  Key limitations of the study include the lack of recent data of TFA intake and the small sample sizes used to estimate intakes in subgroups. |
| At this stage, we ask that you include a short, non-technical Author Summary of your research to make findings accessible to a wide audience that includes both scientists and non-scientists. The Author Summary should immediately follow the Abstract in your revised manuscript. This text is subject to editorial change and should be distinct from the scientific abstract. Please see our author guidelines for more information: <https://journals.plos.org/plosmedicine/s/revising-your-manuscript#loc-author-summary> | We have added an Author Summary as requested. | Page 4:  **Why was this study done?**   - Intake of trans-fatty acids is a well-known risk factor of ischemic heart disease and several countries have implemented strategies with mandatory limits of trans-fatty acids in foods. - In Australia, intake of trans-fatty acids is on average low but does not appear to have decreased in the last decades and is high in certain groups. - A legislative ban to eliminate industrial trans-fatty acids from the Australian food source is a plausible strategy to reduce disease burden and health inequality.   **What did the researchers do and find?**   - We modelled the estimated health effects, costs, cost-effectiveness, and impact on health equality of a nationwide ban of industrial trans-fatty acids in Australia. - Over the lifetime of the adult population, around 40,000 ischemic heart disease deaths could be prevented and about 100,000 health-adjusted life-years could be gained. - The ban was estimated to be cost-saving or highly cost-effective and could reduce health inequalities in the first 10 years after implementation.   **What do these findings mean?**   - A legislative mandatory limit of industrial trans-fatty acid in Australian foods could be a cost-effective strategy to reduce ischemic heart disease and related deaths and healthcare costs. - Socioeconomically disadvantaged groups and Australians outside major cities could potentially have the greatest health gains from such legislation. - Our findings suggest that even in countries like Australia where intake is low, elimination of industrial trans-fatty acids can improve public health and health equality. |
| The Academic Editor for your paper commented that it could be made much clearer in the Discussion how the findings add to or contribute additionally beyond current evidence from other modelling studies. Specifically in the Discussion section 'Interpretation and policy implications' it might be an idea to more explicitly start this section with a summary of what current evidence shows and then go on to clarify how the current analyses add evidence beyond that. | We have revised the discussion to summarize findings from previous modelling studies and how our findings expand the body of evidence. | Page 23, second paragraph:  Previous modelling studies have estimated the impact on cardiovascular disease burden of plausible legal limits or bans of iTFA in the UK [26, 28, 33, 34], and the EU [35]. In all of these studies, such legislative policies were estimated to reduce the number of cardiovascular events or deaths and/or increase HALYs or life years [36]. Three of the studies evaluated the cost-effectiveness of bans or legal limits, taking into consideration health gains, healthcare cost savings, and policy costs [26, 28, 35]. A legal limit of iTFA in the EU (assumed to remove all iTFA) [35] and total bans in the UK [26, 28] were estimated to be cost-saving. Bans of iTFA in the UK were also estimated to reduce health inequalities [26, 28]. An important and novel aspect of our study is that the modelled population already has a low average level of TFA intake (mean:0.59 %E). In previous modelling studies of different strategies to reduce TFA intake in the UK and the EU [26, 37], where average TFA intake is higher (e.g., estimated TFA intake in the UK: 0.79%E [26]), it has been estimated that total bans of iTFA across the food supply would be superior to other evaluated strategies (e.g., voluntary limits and mandatory labelling, or TFA ban targeting restaurant and takeaway food) with regards to health benefits and cost-effectiveness [26, 37]. Our study builds on and significantly extend these prior studies, and suggest legislating to remove iTFA could still lead to significant health benefits and be done in a highly cost-effective way, even in a population with relative low average TFA intake like Australia… |
| Did your study have a prospective protocol or analysis plan? Please state this (either way) early in the Methods section.  a) If a prospective analysis plan (from your funding proposal, IRB or other ethics committee submission, study protocol, or other planning document written before analyzing the data) was used in designing the study, please include the relevant prospectively written document with your revised manuscript as a Supporting Information file to be published alongside your study, and cite it in the Methods section. A legend for this file should be included at the end of your manuscript.   b) If no such document exists, please make sure that the Methods section transparently describes when analyses were planned, and when/why any data-driven changes to analyses took place.    c) In either case, changes in the analysis-- including those made in response to peer review comments-- should be identified as such in the Methods section of the paper, with rationale. | We have attached our internal study proposal to the appendix. We have also revised the methods section to clarify any deviations from this pre-specified plan. | Appendix in supplementary material.  Page 6, first paragraph:  The initial study proposal is presented in the Appendix.  Page 6, third paragraph:  We initially planned to evaluate the health impact of the ban stratified by Aboriginal status (Appendix) but could not reliably estimate age-and-sex-specific intake distributions due to small sample sizes.  Page 9, second paragraph:  We initially planned to use WHO benchmarks for definition of cost-effectiveness (Appendix), but decided to use an Australia-specific definition instead where cost-effectiveness was defined as ICER< AUD 169,361 per HALY gained (i.e., the value of a statistical life year in 2007 inflated to 2010 using consumer price index. |

Table 2. Comments from Reviewer 1

| **Comment** | **Response** | **Modifications made** |
| --- | --- | --- |
| The authors present their modelling study that attempts to estimate the public health impact and cost-effectiveness of a national ban of iTFAs in Australia, considering both policy costs and reduced IHD burden and health care expenditures.   **There are some typos and grammatical errors to be corrected throughout the manuscript. although overall this is a well written and thorough paper.** | Thank you. We have reviewed and corrected typos and grammatical errors in the revised manuscript. | Throughout the manuscript |
| The use of a multiple cohort proportional multi-state life table (Markov) model appears to be appropriate and reasonable in this context. The model formulation provided by the authors looks to be fit for purpose.  The authors have conducted sensitivity analyses, which demonstrate the robustness of the model to many of the key assumptions and parameters.  **Additionally, further strength would be illustrated by including sensitivity analyses surrounding the assumed costs described in the 'Healthcare and policy costs' section.** | We conducted one sensitivity analysis related to policy costs, where we assumed a greater number of products containing iTFA compared to our main analysis. This did have an impact on initial and ongoing reformulation costs as well as repackaging costs. However, given that the industry costs were only a small proportion of total policy costs (especially over the population lifetime), this had little impact on the overall results and cost-effectiveness. We conducted two additional sensitivity analyses to test the impact of policy costs:   1. Given that monitoring costs formed the single greatest cost component, we have now conducted an additional sensitivity analysis assuming that monitoring costs were 25% greater than assumed in the main analysis. Given the differences between the UK (from where our assumption for the main model was based) and Australia (e.g. geographic dispersion), it is possible that monitoring costs maybe greater per capita in Australia compared to the UK. 2. Experience of TFA regulations in Denmark has suggested negligible reformulation costs [1] and thus we conducted a sensitivity analysis assuming no industry costs.   The results of these two sensitivity analyses (i.e., 25% greater monitoring costs or no industry costs), indicated only minor impact on net costs and ICERs regardless of time horizon (Figure 5).  We have incorporated these new analysis into the revised manuscript. | Page 12, last two sentences :  Experience of TFA regulations in Denmark has suggested negligible reformulation costs and thus we conducted a sensitivity analysis assuming no industry costs. Given the differences between the UK (from where our assumption for the primary model was based) and Australia (e.g. geographic dispersion), it is possible that monitoring costs maybe greater per capita in Australia compared to the UK. Thus, we also evaluated the impact of 25% greater monitoring costs compared to our primary model.  Page 21, first paragraph:  Assumptions regarding lower post-ban TFA intake, abundance of iTFA containing products in Australia, and monitoring or industry costs had minor impact on model estimates (Figure 5). |
| "Costs were adjusted to 2010 values, and in the base case analysis, a 3% discount rate was used". **It is noted that a sensitivity analysis of the discount rate from 0-6% has been undertaken by the authors, but can the authors please provide a justification (preferably evidence based) for choosing a discount rate of 3%?** | In line with the recommendations of the first and second panels on cost-effectiveness in health and medicine, we utilized a 3% discount rate in our main analysis [2, 3]. Although the suitability of this rate for cost-effectiveness analyses in low- and middle-income countries is debated, we believe it is a reasonable rate in countries like Australia. However, as the reviewer notes, we did evaluate alternative discount rates, also in line with the recommendations from the 1^st^ and 2^nd^ panels on cost-effectiveness in health and medicine, and found that the policy would be cost-saving over the population life-time when assuming a 6% discount rate. When a 0% discount rate was assumed, the policy was still highly cost-effective in both time horizons.  We have revised the paper to properly cite the 1^st^ and 2^nd^ panels on cost-effectiveness in health and medicine for our assumptions regarding discount rate. | Page 6, first paragraph, last sentence:  Costs were adjusted to 2010 values, and in line with the recommendations of the first and second panels on cost-effectiveness in health and medicine, we used a 3% discount rate in the main analysis. |
| The authors present their study and findings fairly, including uncertainty clearly in their results, figures and conclusions.  In addition to the statistical analyses completed and presented in the results section, did the authors consider different intervention impacts?  Furthermore, what the breakpoint for cost effectiveness is?   I.e what minimum value does the PIF need to be in order for the intervention to be considered cost effective? | Apart from our main analysis, which assumed a post-intervention intake of TFA of 0.5±0.05 %E [4, 5], we conducted a sensitivity analysis assuming a post-intervention intake of 0.4±0.04 %E [6]. This sensitivity analysis represents a scenario where iTFA contributes more to the total TFA, yet most TFA would still come from natural sources.  To further examine the impact of the policy effect on TFA intake, we have now conducted a suite of sensitivity analyses gradually altering the mean TFA intake after the ban from 0.5%E to 0.6%E. As the model utilizes the full distribution (assumed to be lognormal) for the calculation of the PIF, we also alternated the standard deviation of the post-intervention from 10% of the mean (i.e., main model) to 50% of the mean.  We present the results from these sensitivity analyses in Supplementary table 7. While the ICERs increased with increasing mean and standard deviations of post-ban TFA intakes, the ban was still highly cost-effective under any tested assumption of post-intervention TFA intake distributions, with the upper boundaries of the 95% UI not exceeding 20% of the cost-effectiveness threshold specified, i.e., $169,361 AUD per HALY gained. Thus, a legislative elimination of iTFA is likely to be cost-effective, not so much due to a large reduction in population mean intake, but mainly by reducing the intake among individuals with high iTFA intake. | Page 12, last paragraph:  We also evaluated the impact of higher post-intervention intakes (e.g., due to suboptimal compliance to the ban or lower contribution of iTFA to total TFA) with greater mean (0.52-0.60%E) and with standard deviations equal to 10% or 50% of the mean.  Page 21, last two sentences:  Assumptions of a less effective elimination of iTFA (i.e., greater post-ban TFA intake with wider distribution) resulted in increased ICER, especially over the 10-year horizon (Supplementary Table 7). However, the estimated ICER across all such sensitivity analyses were estimated to be <20% of the cost-effectiveness threshold, i.e. the intervention remains highly cost-effective.  Supplementary table 7. |

**Reviewer 2**

Thank you for the opportunity to review PLOS Medicine MS 19-03970, "Estimated health benefits, costs, and cost-effectiveness of eliminating industrial trans- fatty acids in Australia." This paper used a Markov simulation model to estimate the benefits, costs, and cost-effectiveness of a ban on industrial trans-fatty acids in Australia over a 10-year and lifetime time horizon. The authors find that under all scenarios, such a ban would be cost-effective, and under certain assumptions (e.g., 6% discount rate) would be cost-saving. The paper is well-written, well-executed, and speaks to an interesting and important policy question. Main strengths are use of high-quality data sources, evaluation of an important policy question, incorporation of industry costs, and examination of impacts on health disparities. In my read, there are two key limitations, which comprise my major concerns:

Table 3. Comments from Reviewer 2

| **Comment** | **Response** | **Modifications made** |
| --- | --- | --- |
| Major Concern 1: First, the data used to estimate TFA intake are somewhat old now (2011-2012), and there is no discussion of whether TFA intake may have increased or declined since that time. This limitation could be overcome by either citing evidence that TFA has not changed appreciably since 2011-2012, or by varying estimates of TFA intake in sensitivity analyses (e.g., assume average TFA intake is 25% or 50% or 75% of what it was in 2011-2012 across all groups). | This is an important comment. We acknowledge that the infrequent nationally representative surveys on TFA intake in Australia is an issue for nutrition research in Australia, and a limitation of our study.  However, there are multiple strands of evidence suggesting that intake level is very unlikely to have shifted substantially over time.   1. There was no government policy or mandate to reduce iTFA in the last 2 decades [7]. 2. Large survey of food products suggest iTFA levels remained largely similar in food products between 2005 and 2013 [7]. 3. Consistently, this is reflected in stable levels of TFA intake assessed by nationally representative surveys in 1995 and 2011-2012 [8].   This evidence suggest it is reasonable to assume TFA intake levels remain similar between 2011-2012 and now.  We have now expanded the discussion section to acknowledge the lack of more recent intake data as a limitation while recognizing the evidence suggesting fairly stable TFA intake in Australia since the early 2000s.  We also examined the impact of a smaller reduction in TFA intake due to lower pre-intervention intake (i.e., mean intakes lower than estimated in 2011-2012 NNPAS). In this sensitivity analysis we assumed the same post-intervention intake as for the primary model (i.e., 0.5±0.05%E), but lowered the pre-intervention mean intake so that difference between pre and post mean intakes were 50% of the primary model. However, as with all other sensitivity analyses, the ban was estimated to be highly cost-effective. | Page 12, second paragraph:  We also evaluated the impact of lower pre-intervention intakes, with the post-intervention mean intake of the primary model (i.e., 0.50 %E) but only half the difference between the pre- and post-intervention mean intakes.  Page 21, first paragraph:  Assumptions regarding lower pre-or post-intervention TFA intakes; abundance of iTFA containing products in Australia; and monitoring or industry costs had minor impact on model estimates (Figure 5).  Page 25, first paragraph:  The most recent nationally representative assessment of TFA intake was conducted nearly a decade ago and iTFA intake may have changed. However, there has been no government policy or mandate to reduce iTFA in the last two decades [10], iTFA content in food products has been largely similar between 2005 and 2013 [10], and TFA intake remained stable from 1995 to 2010-2011 [4]. This suggest that it is reasonable to assume fairly stable TFA intake levels since 2011-2012. |
| Major Concern 2: Second, there is no discussion of how companies are likely to reformulate when they remove TFA. If companies increase saturated fat when removing TFA, or add more sodium or sugar, this might offset some of the benefits of lower TFA on IHD incidence and death. This needs to be explored to paint an accurate picture of the effects of TFA on health. | Thank you for this comment. We agree with the reviewer that the potential impact of food reformulations depends on the substitute for the targeted nutrient.  We used data from a meta-analysis directly linking TFA intake with incident coronary heart disease, which removes the uncertainty of modelling through the mediating effects of cholesterol, and it includes non-cholesterol mediating effects such as via inflammation.  Importantly, the relative risk associated with a change in TFA intake is not specific for a certain substitute, but rather it contrasts the TFA intake against the overall diet. It is true that if iTFA are replaced by cis-isomers of unsaturated fatty acids, the potential health gains could be greater than if the replacement would constitute of palmitic acid or other saturated fatty acids.  Recent comprehensive review of the literature suggest that reformulation to decrease or eliminate TFA from foods was not linked to any discernible patterns of systematic replacement by saturated or polyunsaturated fats [9]. This suggest that our modelling of iTFA removal against an overall diet is a reasonable approach.  We have revised our discussion to clarify that:   1. Consistent with prior modelling papers, our model assesses change in TFA against the overall diet rather than specific substitution with other types of fats. 2. Evaluations of product reformulation has not identified consistent patterns of increase or decrease in other types of dietary fats as replacement for iTFA. | Page 25, second paragraph:  Consistent with prior modelling papers, our model uses risk estimates of change in TFA against the overall diet rather than specific substitution with other types of fats. If the iTFA would be systematically replaced by saturated fatty acids, the impact of the ban could be lower than estimated here. However, evidence suggests no overall increase in saturated fatty acid content in food products after previous reductions of iTFA [10]. |
| If these concerns can be addressed, I believe this manuscript would make an important contribute to the literature and would be appropriate for publication in PLOS Medicine.  My other comments/suggestions are minor.  Abstract: 1.      Need to define "iTFA" here. 2.      I would suggest you state somewhere in the abstract the policy you're modeling - i.e., a TFA ban. Otherwise, it's not clear what "policy costs" are, since no policy has been mentioned. | 1. We have defined iTFA as suggested 2. We have revised the abstract to clarify that we modelled a ban. | Abstract:  Elimination of industrial TFA (iTFA) from the Australian food supply could result in reduced IHD mortality and morbidity while improving health equity.  Thus, we assessed the potential cost-effectiveness, health gains, and effects on health equality of an iTFA ban from the Australian food supply. |
| Introduction 3.      "1% E" - I would spell out energy since you have not defined this abbreviation yet. (I would personally prefer "energy" to "E" throughout, but at very least the abbreviation needs to be defined at first use).  4.      Noted above, but -- are there more recent data on TFA since 2013? It's now almost 7 years later, so it seems possible that there would be additional reductions in TFA in the Australian food supply since 2013, particularly given that other countries have adopted policies since then (so multi-national corporations might reformulate products to, e.g., comply w/ US regulations, even if some of those products are sold in the US). 5.      "Our prior modelling suggest at current level of intake, TFA continues to contribute to around 500 deaths per year…" I would clarify this is among Australians.   6.      Would be nice to note whether Australia has considered this policy. (The simulation is useful even if the policy has not been proposed yet, but if it has, would be good to note it's on policymakers' radar).  7.      You might mention here why it's useful to conduct this simulation given Australia's low levels of TFA intake. I really liked this section of the discussion and think including a brief mention of these points in the intro might help motivate the paper, particularly for readers who think we already know what we need to know about TFA bans. | 3. We have defined the abbreviation for energy percentage as suggested.  4. As noted above, there is little evidence to suggest that TFA intake would have reduced since the most recent dietary survey in 2010-2013: a) no government policy to reduce iTFA over the last 2 decades, b) no noticeable change in TFA content between 2005-2013, b) no decreasing trend in TFA intake between 1995 and 2011-2012.  5. We have clarified this as suggested.  6. To our knowledge, Australia has so far not considered a ban of iTFA.  7. Thank you for this comment. We have revised the introduction to highlight the potential health benefits of a ban on iTFA even in countries like Australia where the intake is low. | 3. Page 4, “Introduction”, second paragraph, first sentence:  Given the adverse effects of TFAs, the World Health Organization (WHO) recommends limiting TFA intake to 1 energy percent (%E) and countries have implemented strategies to reduce industrial derived TFA (iTFA) in the food supply such as partially hydrogenated vegetable oils [5, 6].  5. Page 5, first paragraph, last sentence:  Our prior modelling suggests that at current levels of intake, TFA continues to contribute to around 500 deaths per year in Australia, with the majority of these expected to accrue to those with less education and income.  6. Page 5, first paragraph, last sentence To our knowledge, Australia has so far not considered a ban of iTFA. In order to further inform policy regulations related to iTFA in Australia, we conducted a modelling study that estimated the public health impact and cost-effectiveness of a national ban of iTFAs in Australia, considering both policy costs and reduced IHD burden and health care expenditures.  7. Page 5, second paragraph, second sentence:  Despite being comparatively low, the uneven distribution of consumption means that a part of the population consumes TFA in harmful quantities and TFA intake levels in Australia continue to contribute to the IHD burden [12]. Further removal of iTFA from the food supply through mandatory limits (iTFA ban) could have additional benefits in Australia and other countries with similarly low TFA intake but no mandatory limits of iTFA content in foods. |
| Methods 8.      I commend the pre-specification of the subgroups. Is there an associated protocol that describes the pre-specified analyses? If so, would be helpful to include it as a hyperlink or appendix file.  9.      Intervention: It would be good to clarify whether the ban applies to all foods or just packaged foods. Later you notice that the FoodSwitch database does not contain restaurant foods, so I assume the ban would apply to all foods, but good to clarify. 10.     Study design: Here or in the introduction, you might give readers a sense of why these subpopulations are meaningful. The Socio-Economic Disadvantage groups are intuitively meaningful, but for non-Australian readers, it's not immediately clear why it matters to examine remoteness. 11. Can you clarify the sample size of the Australian National Nutrition and PA Survey? Was sample size adequate to calculate reliable mean TFA intake for all age and sex groups?  12.     Data sources. In locations where TFA has been banned, is it true that intakes drop to <= 0.5% energy intake? That is, do bans effectively remove all iTFA or do some companies continue to include some TFA in their products? I would state the evidence for this, or vary the assumption in sensitivity analyses.  13.     Data sources: It wasn't clear to me why the intervention would lead to additional annual cost of 1% of the initial reformulation cost - can you clarify? | 8. As noted above, we have uploaded our study proposal that summarize the framework of our modelling and specifies the subgroups to evaluate. In addition, we have revised the Methods section to clarify when and why we deviated from the pre-specified plan.  9. We have clarified that the ban would target both packaged and restaurant foods.  10. We have clarified in the Introduction why remoteness is an important factor for health equity in the Australian context.  11. Supplementary Table 1 contains information about sample size for each sex-age-SEIFA/Remoteness stratum. We have extended the discussion to acknowledge the limitation of small sample sizes especially in the subgroup analyses.  12. We have clarified the assumption regarding the post-ban TFA intake and conducted additional sensitivity analyses alternating this assumption.  13. In line with previous modelling studies [1, 6], we assumed an ongoing annual industry cost of 1% of the initial reformulation costs to conservatively account for reduced industry profits. We have clarified this in the manuscript. Still, reformulation costs had a minor impact on policy and net costs and thus alterations in this assumption would not reasonably change our conclusion that the ban would be cost-effective. | 8. Appendix  9. Methods, first paragraph, first sentence:  Intervention - A complete ban of iTFAs from the Australian food supply. The ban would cover all foods and ingredients containing iTFA and therefore target both packaged and restaurant foods.  10. Introduction, last paragraph, second to last sentence:  Apart from differences across socioeconomic groups, we estimated benefits separately in groups based on remoteness, given that Australians outside of metropolitan areas often have higher disease burden and poorer access to healthcare and diet compared to those living in major cities.  11. Page 25, first paragraph, last sentence:  The sample size of some age-and-sex groups was small (e.g., Australian outside major cities and inner regional areas), which may have resulted in imprecisely estimated means and standard deviations of TFA intake, especially in the subgroup analyses.  12. Page 7, first paragraph, last two sentences:  A mandatory ban in Denmark, virtually eliminated iTFA from the food source [21]. Thus, we assumed the post-intervention TFA intake of all sex-age groups to be equal to the theoretical minimum risk distribution, i.e., 0.50±0.05%E in our primary model (Table 1), based on the likely average intake of non-industrial derived TFAs (i.e., from meat and dairy from ruminant animals).  13. Page 8, first paragraph, last sentence:  In line with previous modelling studies, we assumed an ongoing annual industry cost of equalling 1% of the initial reformulation cost to conservatively account for reduced industry profits. |
| Results 14.     Figure 1. This is a helpful visual. You might consider modifying to use four panels and separating the deaths from the incidences (vs. the current two panels that use different colored bars to show deaths vs. incidences). This might make it easier to see the pattern of results, particularly for SEIFA (where the white bars are small and somewhat overwhelmed by the gray bars). A similar comment would apply to the other tables using this design, but I found Figure 1 most difficult to discern.   15.     Figure 5. This was hard to follow. Consider using color coding to show the different models, instead of the arrows/lines. | 14. We have revised Figures 1-3 as suggested.  15. We have replaced Figure 5 with a figure of the cost-effectiveness planes with estimates and summary statistics of the primary model and deterministic sensitivity analyses depicted in diverse colours. | Figures 1-3 and 5. |
| Discussion 16.     I appreciate that the study used the most recent data on TFA intake, but these data are relatively outdated now. It would be good to note this as a limitation, particularly if the authors are unable to locate more recent data on trends of TFA content since 2013.  17.     The introduction stated that the authors' previous work has found that TFA contributes to about 500 death/year. The present work suggests banning iTFA would avert about 2,300 IHD deaths over 10 years (about 230/year). I assume the discrepancy is either due to modeling differences or because the 500/year statistic includes non-IHD deaths due to TFA. Consider clarifying/comparing? | 16. As mentioned above, we have extended the discussion to acknowledge the limitation of infrequent nationally-representative dietary assessments in Australia.  17. The difference is due to the previous model going straight from TFA consumption levels to mortality, without lag time. In the new, expanded version of the model, TFA consumption levels impact on disease incidence, which leads to lower prevalence in the years after, and mortality is a function of prevalence. After 10 years, one would only see the beginning of a rising benefit in terms of mortality. | Page 25, first paragraph:  The most recent nationally representative assessment of TFA intake was conducted nearly a decade ago and thus, iTFA intake may have changed. However, there has been no government policy or mandate to reduce iTFA in the last two decades [10], iTFA content has been largely similar in food products between 2005 and 2013 [10], and consequently TFA intake remained stable between 1995 to 2010-2011 [4]. Hence, this suggest that it is reasonable to assume fairly stable TFA intake levels since 2011-2012. |

**Refereneces**

1.	Pearson-Stuttard, J., et al., *Cost-effectiveness analysis of eliminating industrial and all trans fats in England and Wales: modelling study.* J Public Health (Oxf), 2017. **39**(3): p. 574-582.

2.	Weinstein, M.C., et al., *Recommendations of the Panel on Cost-Effectiveness in Health and Medicine.* JAMA, 1996. **276**(15): p. 1253-1258.

3.	Sanders, G.D., et al., *Recommendations for Conduct, Methodological Practices, and Reporting of Cost-effectiveness Analyses: Second Panel on Cost-Effectiveness in Health and Medicine.* Jama, 2016. **316**(10): p. 1093-103.

4.	Wang, Q., et al., *Impact of Nonoptimal Intakes of Saturated, Polyunsaturated, and Trans Fat on Global Burdens of Coronary Heart Disease.* J Am Heart Assoc, 2016. **5**(1).

5.	Wu, J.H.Y., et al., *Contribution of Trans-Fatty Acid Intake to Coronary Heart Disease Burden in Australia: A Modelling Study.* 2017. **9**(1): p. 77.

6.	Allen, K., et al., *Potential of trans fats policies to reduce socioeconomic inequalities in mortality from coronary heart disease in England: cost effectiveness modelling study.* BMJ, 2015. **351**: p. h4583.

7.	Wu, J., et al., *Levels of trans fats in the food supply and population consumption in Australia: an Expert Commentary rapid review brokered by the Sax Institute (*[*www.saxinstitute.org.au*](www.saxinstitute.org.au)*) for The National Heart Foundation of Australia*. 2017.

8.	Wanders, A.J., P.L. Zock, and I.A. Brouwer, *Trans Fat Intake and Its Dietary Sources in General Populations Worldwide: A Systematic Review.* Nutrients, 2017. **9**(8): p. 840.

9.	Downs, S.M., et al., *The Impact of Policies to Reduce trans Fat Consumption: A Systematic Review of the Evidence.* Current Developments in Nutrition, 2017. **1**(12).
