# Supplementary material for: Estimated health benefits, costs, and cost-effectiveness of eliminating industrial trans-fatty acids in Australia: A modelling study
Source: PLoS Med. 2020 Nov 2;17(11):e1003407. doi: 10.1371/journal.pmed.1003407 (PMC7605626; doi:10.1371/journal.pmed.1003407)
Supplement: Attachment — Submitted filename: Response to editor queries - September 15.docx [file pmed.1003407.s011.docx]

Dear editor,

We are grateful for this opportunity to revise our manuscript and we hope that the requested changes made (**Table**) are satisfactory and the manuscript could now be accepted for publication in Plos Med. We also want to bring to your attention some additional revisions that we have made:

While the revised version of this manuscript submitted in June was under review, we published an updated analysis of the abundance of iTFA-containing products in the Australian food supply (Huang et al. 2020). That analysis revealed 131 food products with ingredient lists indicative of iTFA, compared to the 122 products we identified in our initial analyses used in the current study to estimate reformulation and repackaging costs. Thus, we have updated the current version of our manuscript with the slightly higher number of iTFA containing products. However, as reformulation and repackaging costs corresponds to a very minor fraction of the total net costs estimated from the ban, these updated analyses had very minor impact on our model estimates and did not in any way impact our conclusions. We revised Table 2, Figure 5, and Supplementary Table 7 in response to these updated analyses and updated the manuscript text as follows (changes in red):

**Methods / “Healthcare and policy costs”**

*We estimated this number by identifying products in the 2018 Australian FoodSwitch database [27] that contained any terms indicative of iTFA in the ingredient list (i.e., “partially hydrogenated fat”, “hydrogenated vegetable oil”, or “hydrogenated”) [28]. Of 28,349 foods included in the analysis, 131 products (0.5%) contained specific ingredients indicative of iTFA [28].*

**Results / “Overall health gains, costs and cost-effectiveness”**

*Banning and elimination of iTFA from the Australia food supply, was estimated to cost nearly AUD 22 million during the first 10 years and AUD 56 million over the population life time (Table 2), with a majority of estimated costs attributed to government costs for monitoring.*

…

*Thus, the net policy costs over 10 years and the population lifetime were estimated as AUD 7.2 million and 212.9 million, respectively (Table 2). The elimination of iTFA in Australia was estimated to be cost-saving (i.e., net cost<0 AUD) to highly cost-effective during the first 10 years (1,153 AUD/HALY [95% UI: dominant; 3,578], i.e., ≤ 2% of the value of a statistical life year) and highly cost-effective over the population life time (1,961 AUD/HALY [95% UI: 1,015; 2,756], Table 2).*

**Results / “Sensitivity analyses”**

*The greatest ICER during the first ten year was estimated when assuming 0% discount rate: 3,308 AUD/HALY (95% UI: dominant; 5,970). Assuming the same discount rate over the population life time also suggested that the ban would be highly cost effective, with estimated ICER 15,423 (95% UI: 14,176; 16,540).*

Reference:

Huang, L., Federico, E., Jones, A. and Wu, J.H. (2020), Presence of trans fatty acids containing ingredients in pre‐packaged foods in Australia in 2018. Australian and New Zealand Journal of Public Health. doi:10.1111/1753-6405.13014

**Note:** The reference above has been added to the manuscript as reference #28.

**Table.** Comments to queries from editors

| Comment | Response | Revision made |
| --- | --- | --- |
| Title- please can you add “a” to the study descriptor to read “a modelling study” | We have revised the title as suggested. | Title |
| Throughout- please add punctuation after the reference square brackets please | We have made the requested revisions. | Throughout manuscript |
| Throughout the manuscript, whenever you restate (eg at the start of the Discussion) the estimated deaths or cases averted, it should be clear what you are comparing to (the current level of TFA intake). | We have made the requested revisions throughout the Abstract and the Results and Discussion sections. | Abstract/”Methods and Findings”: 7^th^ sentence  Results/”Overall health gains, costs and cost-effectiveness”: 1^st^ sentence  Results/  Discussion/First paragraph: 1^st^ sentence |
| Rephrase the Abstract conclusions to include "Our model estimates that..." or similar language. Also could include more on the implications, using similarly cautious language. The last point of the Author Summary could perhaps be retooled for the Abstract Conclusions. Please use similar cautious language in the discussion | We have made the requested revisions in the Abstract, the Author summary and the Discussion. | Abstract/Conclusion: 1^st^ sentence  Author summary/Third section: Last sentence |
| Page 5- “Generally Recognized as Safe for..” please consider if these words need to be capitalised? | We have revised as suggested to “… generally recognized as safe for “ | Introduction/Second paragraph: 3^rd^ sentence  Discussion/” Interpretation and policy implications”: 5^th^ sentence from end |
| Page 13 “As there have been suggested that…” seems to have been cut off Please ensure that the study is reported according to the CHEERS guideline, and include the completed checklist as Supporting Information. When completing the checklist, please use section and paragraph numbers, rather than page numbers. Please add the following statement, or similar, to the Methods: "This study is reported as per the xxx guideline (S1 Checklist)." | We have deleted the words “As there have been suggested that…” that were accidently included in the previous version of the manuscript.  We have completed and included a CHEERS checklist in the supplementary material. | Methods/”Statistical analysis”  S1 Checklist  Methods/First paragraph |
| Methods- please remove the sub-heading “Intervention” as it is a bit misleading for a modelling study. I recommend paraphrasing the sentence to include the aim of your work including the study design used. | We have revised the Methods section as suggested. | Methods/”Study design”: 1^st^ sentence |
| Methods-please provide further information about the cohorts used in the study. These are very briefly mentioned and it would help to outline the participant demographics in the methods. | We have revised the Method subsection “Health outcomes” to include more information of the total and subgroup-specific model populations. | Methods/“Health outcomes” |
| Please use table footnotes to add explanations for acronyms | We have revised the table footnotes to consistently explain acronyms and abbreviations. | Tables 1-3 |
| Discussion- I’m not sure what you mean by “population lifetime” could you please clarify in text? | We have added the following definition to the first mentioning in the Methods, Results, and Discussion sections:  The time from policy implementation until all individuals died or reached 100 years of age. | Methods/”Study design”: 3^rd^ sentence from end  Results/”Overall health gains, costs and cost-effectiveness”: 1^st^ sentence  Discussion/First paragraph: 2^nd^ sentence |
| Please start the discussion with a 1-2 sentence summary of what was done before delving into the main findings | We have added the following sentence to the beginning of the discussion: *We used national representative data in Markov cohort models to estimate the impact on IHD burden and health equity, as well as the cost-effectiveness of a national ban of iTFA in Australia compared to no ban and current levels of TFA intake.* | Discussion/1^st^ paragraph: 1^st^ sentence |
| I think it is important to distinguish between cost saving and cost effectiveness here- since you aimed to look at cost effectiveness. Please use standard terminology throughout the submission to avoid confusion | We have revised the manuscript to clarify the definitions of cost-saving (i.e., negative net cost) and cost-effective (i.e., ICER less than the value of a statistical life year per HALY). | Author summary/ 2^nd^ paragraph: last sentence  Methods/”Statistical analysis”/”Estimation of health benefits and cost-effectiveness”: last two sentences  Results/” Overall health gains, costs and cost-effectiveness”: last sentence |
| Please ensure all acronyms are introduced on first view | We have revised the manuscript as suggested | Throughout manuscript |
| Please use Vancouver style for the references and remove all iterations of [Internet] from the reference list | We have revised the reference list as suggested. | Reference list |
| Data availability statement- please mention the data sources used in the study and provide the code used to undertake analyses in a suitable repository. | We have added the data availability statement to the end of the manuscript:  “All relevant data are within the paper and its Supporting information files”. | Data availability statement |
